# Tick‐borne pathogens, including Crimean‐Congo haemorrhagic fever virus, at livestock markets and slaughterhouses in western Kenya

**DOI:** 10.1111/tbed.13911

**Published:** 2020-12-05

**Authors:** Tatenda Chiuya, Daniel K. Masiga, Laura C. Falzon, Armanda D. S. Bastos, Eric M. Fèvre, Jandouwe Villinger

**Affiliations:** ^1^ International Centre of Insect Physiology and Ecology (icipe) Nairobi Kenya; ^2^ Department of Zoology and Entomology University of Pretoria Pretoria South Africa; ^3^ Institute of Infection, Veterinary and Ecological Sciences University of Liverpool Neston UK; ^4^ International Livestock Research Institute Nairobi Kenya

**Keywords:** East Africa, emerging infectious disease, *Nairovirus*, *Rhipicephalus*, *Rickettsia*, Zoonoses

## Abstract

Vectors of emerging infectious diseases have expanded their distributional ranges in recent decades due to increased global travel, trade connectivity and climate change. Transboundary range shifts, arising from the continuous movement of humans and livestock across borders, are of particular disease control concern. Several tick‐borne diseases are known to circulate between eastern Uganda and the western counties of Kenya, with one fatal case of Crimean‐Congo haemorrhagic fever (CCHF) reported in 2000 in western Kenya. Recent reports of CCHF in Uganda have highlighted the risk of cross‐border disease translocation and the importance of establishing inter‐epidemic, early warning systems to detect possible outbreaks. We therefore carried out surveillance of tick‐borne zoonotic pathogens at livestock markets and slaughterhouses in three counties of western Kenya that neighbour Uganda. Ticks and other ectoparasites were collected from livestock and identified using morphological keys. The two most frequently sampled tick species were *Rhipicephalus decoloratus* (35%) and *Amblyomma variegatum* (30%); *Ctenocephalides felis* fleas and *Haematopinus suis* lice were also present. In total, 486 ticks, lice and fleas were screened for pathogen presence using established molecular workflows incorporating high‐resolution melting analysis and identified through sequencing of PCR products. We detected CCHF virus in *Rh. decoloratus* and *Rhipicephalus* sp. cattle ticks, and 82 of 96 pools of *Am. variegatum* were positive for *Rickettsia africae*. Apicomplexan protozoa and bacteria of veterinary importance, such as *Theileria parva*, *Babesia bigemina* and *Anaplasma marginale*, were primarily detected in rhipicephaline ticks. Our findings show the presence of several pathogens of public health and veterinary importance in ticks from livestock at livestock markets and slaughterhouses in western Kenya. Confirmation of CCHF virus, a *Nairovirus* that causes haemorrhagic fever with a high case fatality rate in humans, highlights the risk of under‐diagnosed zoonotic diseases and calls for continuous surveillance and the development of preventative measures.

## 1 INTRODUCTION

Ticks are vectors of a range of viral, bacterial and protozoal pathogens that cause huge economic losses to livestock farming communities, while some are zoonotic and as a consequence present a public health burden (de la Fuente et al., 2008). Among the most prevalent livestock diseases in Kenya are babesiosis, theileriosis and anaplasmosis (Franck et al., 2015; Gachohi et al., 2012; Latib et al., 1995; Norval et al., 1984), while zoonotic rickettsiosis constitutes a serious emerging public health threat globally (Brown & Macaluso, 2016; Fournier et al., 2017; Jensenius et al., 2017; Maina et al., 2017; Ndip, Bouyer, et al., 2004; Ndip, Parola et al., 2013; Rutherford et al., 2004). In addition to *Rickettsia*, tick‐borne bacteria such as *Ehrlichia* and *Anaplasma* and protozoa such as *Babesia* have been shown to infect humans in the Americas and Europe (Doudier et al., 2010). Ticks also transmit nairoviruses, most of which cause a mild non‐pathognomonic febrile illness in humans, but some, such as Crimean‐Congo haemorrhagic fever (CCHF) and Dugbe viruses, can cause severe systemic illness and mortality, affirming the importance of ticks in the transmission of viral haemorrhagic fevers (Papa et al., 2017). In livestock, Nairobi sheep disease virus, also a *Nairovirus*, is a constant threat to sheep production in East Africa and the Horn of Africa (Baron & Holzer, 2015).

With travel and trade thought to be major drivers of emerging pathogen spread (Kilpatrick & Randolph, 2012), the movement of livestock and people among East African countries could enhance the circulation of emerging pathogens, especially given that high arboviral activity has been reported across the region (Mossel et al., 2017; Nyaruaba et al., 2019). Smallholder livestock production in East Africa is associated with livestock movement across provincial and national borders to livestock markets in peri‐urban areas (Fèvre et al., 2005) in which animals have been found to be heavily infested by ticks (Sang et al., 2006). Livestock movement plays a major role in the introduction of infective foci in naïve areas where they can then be disseminated by capable vectors (Fèvre et al., 2006). Indeed, livestock movements have been implicated in both past and recent Rift Valley fever (RVF) outbreaks in Kenya (Baba et al., 2016; Munyua et al., 2010; WHO, 2018).

Outbreaks of CCHF (Dunster et al., 2002) and RVF (WHO, 2018) have previously been reported in western Kenya, and there is serological evidence of circulation of chikungunya, yellow fever, West Nile and RVF viruses (Cook, Grossi‐Soyster, et al., 2017; Inziani et al., 2020; Mease et al., 2011; Nyaruaba et al., 2019). While reports on the occurrence of zoonotic vector‐borne bacteria are scant, the high prevalence of malaria in western Kenya results in under‐investigation of other causes of febrile illnesses. Ticks, fleas and lice may be both vectors and reservoirs of most pathogens they transmit, making them an important component in the transmission dynamics of vector‐borne zoonoses (Raoult & Roux, 1997).

Several bacterial pathogens of zoonotic and veterinary potential in ticks have been reported in East Africa, including tick‐borne spotted fever group (SFG) rickettsiosis agents (*Rickettsia africae*, *Rickettsia conorii* and *Rickettsia aeschlimanii*) (Kumsa et al., 2015; Macaluso et al., 2003; Maina et al., 2014; Mwamuye et al., 2017; Nakao et al., 2013; Nakayima et al., 2014). A broad spectrum of bacteria and protozoa of veterinary and public health importance have also been detected, including *Theileria parva*, *Ehrlichia ruminantium*, *Ehrlichia chaffeensis*, *Anaplasma marginale*, *Anaplasma phagocytophilum* and *Anaplasma platys* (Mwamuye et al., 2017; Omondi et al., 2017; Oundo et al., 2020; Ringo et al., 2018; Teshale et al., 2015). *Hyalomma*, *Amblyomma* and *Rhipicephalus* ticks sampled from livestock in North Eastern Kenya were previously shown to be infected with CCHF, Bunyamwera, Dugbe, Ndumu, Semliki forest, Thogoto, Ngari, Dhori and West Nile viruses (Lutomiah et al., 2014; Lwande et al., 2013; Sang et al., 2006, 2011). These viruses are endemic in East Africa (Nyaruaba et al., 2019), and some, such as Semliki Forest, Wesselsbron, Ngari and Bunyamwera viruses, have only been isolated from mosquitoes (Ajamma et al., 2018; Lwande et al., 2013; Villinger et al., 2017). In most instances, ticks with arboviruses were collected from cattle at livestock markets and abattoirs, highlighting the need to carry out surveillance for arboviruses at such facilities.

Flea‐borne rickettsioses, such as flea‐borne spotted fever (*Rickettsia felis*) and murine typhus (*Rickettsia typhi*), both endemic in East Africa, are transmitted by *Ctenocephalides felis* and *Xenopsylla cheopis* fleas, respectively. *Rickettsia felis* and *Rickettsia asembonensis* sp. nov. have been detected not only in *C. felis* (Jiang et al., 2013; Maina et al., 2019), but also in several other flea species (Luce‐fedrow et al., 2015). Louse infestations result in severe pruritic mange in livestock, leading to production losses (Hornok et al., 2010), and epidemic typhus, caused by *Rickettsia prowazekii*, in humans, especially in overcrowded and poor social settings (Raoult & Roux, 1997). While the vectorial capacity of ticks is established, the role of lice and fleas in the epidemiology of vector‐borne zoonoses is rarely investigated.

Active surveillance for zoonotic pathogens and their vectors generates information on their presence and prevalence and can identify novel vector–pathogen associations. Such information can facilitate early detection and quantification of pathogen burdens and thus is important for planning control strategies to reduce spill‐over infection from livestock to humans. Most of the diseases are characterized by non‐specific febrile illness, which can be easily confused with other fever‐causing agents. Awareness of their presence improves clinical referral and diagnosis. Therefore, we carried out this study to investigate the disease risk posed by the movement of tick, flea and louse infested animals via livestock markets (LM)s and slaughterhouses (SHs) in the Lake Victoria basin of East Africa.

## 2 MATERIALS AND METHODS

### 2.1 Study site

The study was carried out in neighbouring counties, viz. Busia, Bungoma and Kakamega, in western Kenya. This region, part of which shares borders with Uganda, is representative of the larger Lake Victoria basin ecosystem and has the highest rural human and livestock population densities in East Africa. The predominant farming type is a mixed smallholder livestock production system, though husbandry practices are rapidly changing as production moves from largely subsistence to increasing intensification, with consequent impacts on disease emergence and transmission (Fèvre et al., 2017).

### 2.2 Study design and sample collection

**FIGURE 1:**
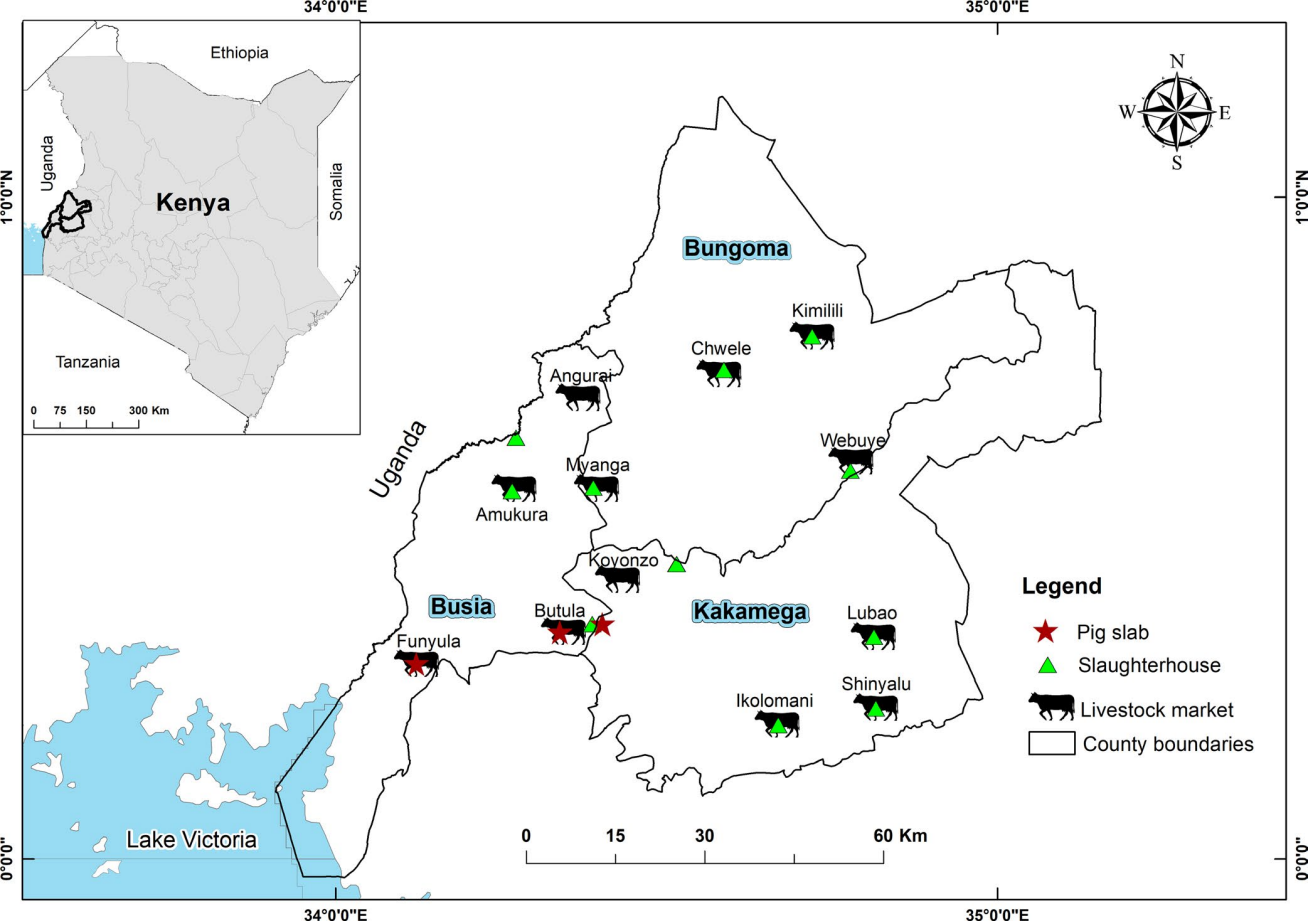
Map of the three neighbouring counties of Busia, Bungoma and Kakamega showing the livestock markets and slaughterhouses from which arthropod samples were collected [Colour figure can be viewed at wileyonlinelibrary.com]

The study design and sampling collection are described in detail elsewhere (Falzon et al., 2019). Briefly, four LMs and neighbouring SHs were selected in each county (Figure 1), where each LM was closely associated with a ruminant or pig SH. At each LM, up to 10 animals were selected via systematic random sampling. During each visit, we attempted to select six to seven cattle and three to four small ruminants, so as to proportionally represent the livestock species present at the LM; no pigs were present at the LMs. Signed consent was sought from the animal owners or traders accompanying sampled animals, and a short questionnaire was administered to capture demographic and animal ownership details. Animals were then physically restrained and, after a general clinical examination, blood was drawn by a qualified veterinarian from the jugular vein using a vacutainer tube. Nasal swabs and faecal samples were also collected. Any external parasites present on the hide of the selected animals were removed with gloved hands and placed into falcon tubes containing 70% ethanol to preserve their morphology for identification purposes (Estrada‐Peña et al., 2004).

At ruminant and pig SHs, we sampled all the animals brought for slaughter (if <10 animals were slaughtered), or a sub‐sample of these (if >10 animals were slaughtered), on a given visit. A similar sampling procedure as at LMs was followed and, in addition to ticks, lice and fleas were also collected if present on sampled animals. The number of animals sampled per visit at each LM and SH was predetermined by the number of samples required for the entire integrated surveillance study and logistical constraints (Falzon et al., 2019). Sample bottles and blood tubes were barcoded and transported to the field laboratory in Busia in a cool box with ice packs. Arthropods were stored at −40°C at the International Livestock Research Institute (ILRI) Department of Veterinary Services laboratory in Busia before being shipped on dry ice to the Martin Lüscher Emerging Infectious Disease (ML‐EID) laboratory at the International Centre of Insect Physiology and Ecology (*icipe*) where they were stored at −80°C for further identification and laboratory analysis.

### 2.3 Morphological identification of ticks, lice, and fleas

Ticks, lice and fleas were morphologically identified to species level using a stereomicroscope (Zeiss) with the aid of identification keys (Centers for Disease Control & Prevention, 2003; Pratt & Wiseman, 1962; Estrada‐Peña et al., 2004). Excessively engorged tick specimens were excluded from the analysis due to difficulties in identifying them and the PCR inhibitory potential of their blood meals (Sparagano et al., 1999). For ticks and lice, the most important morphological features used for identification were body conformation, mouthparts, scutum ornamentation and anal shields. Fleas were identified based on the shape of the head and the arrangement of pronotal and genal combs. Representative specimens were photographed using an Axio‐cam ERc 5s digital camera (Zeiss) mounted on a stereomicroscope. Ticks, lice and fleas were pooled (1–3) according to developmental stage, sex, species and host from which they were sampled.

### 2.4 Nucleic acid extraction from arthropods and selected livestock blood samples

Arthropod pools were homogenized before nucleic acid extraction. Each pool was placed in a 1.5‐ml Eppendorf tube with pre‐weighed scoops of 750 mg of 2.0‐mm and 150 mg of 0.1‐mm yttria‐stabilized zirconium oxide (zirconia/yttria) beads (Biospec), in which they were mechanically disrupted using a Mini‐Beadbeater‐16 (BioSpec) for 60–90 s. Phosphate‐buffered saline (PBS) (360 µl) was added to each tube and vortexed, and 210 µl of the resulting homogenate was transferred to a 96‐well specimen processing cartridge. DNA and RNA were extracted using a MagNA 96 DNA and Viral NA Small Volume Kit (Roche Applied Science) in a MagNA Pure 96 robot (Roche Molecular Systems). A sindbis virus culture isolate was included as a positive extraction control, and PBS was used as a negative extraction control in each run. Total nucleic acid was eluted in 50 µl of RNAse‐free water.

Animal blood samples associated with arthropod pools identified as positive for *R. africae* and CCHF virus were selected for pathogen screening. Nucleic acids from blood samples were extracted using the magnetic bead‐based High Prep Viral DNA/RNA kit (MagBio Genomics). First, 200 µl of blood was added to 1.5‐µl Eppendorf tubes containing 528 µl of a lysis master mix consisting of VDR lysis buffer, isopropanol and carrier RNA and vortexed. Then, 10 µl of proteinase K and 10 µl of MAG‐S1 magnetic beads were added and mixed into solution by inversion. The subsequent steps were performed according to the manufacturer's instructions.

### 2.5 Molecular identification of ticks

While morphology alone was adequate for definitive identification of fleas and lice, we supplemented morphological identification of ticks with DNA sequence identification of 15 single specimens for which morphologic identification to species level was equivocal. Using taxon‐specific primers, we amplified three target genes: the internal transcribed spacer‐2 (ITS2) (Chitimia et al., 2009), cytochrome oxidase 1 (CO1) (Hebert et al., 2004) and 16S ribosomal (r)RNA (Brahma et al., 2014) (Table S1). The PCRs were performed in a SimpliAmp PCR Thermal Cycler (Applied Biosystems, Singapore) in 10‐µl reactions that consisted of 2 µl of 5× HOT FIREPol^®^ Blend Master Mix (Solis BioDyne), 2 µl of template and 0.5 µl of 10 µM primer. Molecular grade water was included as a negative control on each run. The cycling conditions have been described before in detail (Mwamuye et al., 2017), with the exception that the final extension step for the three fragments was seven minutes. Amplicons of the correct size were visualized alongside Quick‐Load^®^ 100‐bp DNA Ladder (Biolabs) by electrophoresis on 1.6% ethidium bromide‐stained agarose gels under UV light. Bidirectional sequencing of amplicons purified by Exo 1‐rSAP combination (Biolabs) was performed by Macrogen. Sequence chromatograms were inspected, edited and aligned using Geneious Prime version 2019.0.4 software (Biomatters). The resulting sequence contigs were used in nucleotide BLAST searches (Altschul et al., 1990) against the GenBank nr database (www.ncbi.nlm.nih.gov/blast) to identify tick species‐specific sequence matches.

### 2.6 Molecular detection of arboviral, bacterial, protozoan pathogens

#### 2.6.1 Detection of arboviruses

A previously described multiplex reverse transcription (RT)‐PCR‐HRM test was initially utilized for the detection of arboviruses within the *Flavivirus*, *Alphavirus*, *Nairovirus*, *Phlebovirus*, *Orthobunyavirus* and *Thogotovirus* genera (Villinger et al., 2017) (Table S1). This was preceded by cDNA synthesis using the High Capacity cDNA Reverse Transcription (RT) kit (Applied Biosystems) in a 20‐µl reaction mixture that contained 10 µl nucleic acid extract, 1 U/μl RNase inhibitor, 100 mM dNTPs, 1× RT buffer, 2.5 u/µl reverse transcriptase enzyme and 40 u/µl non‐ribosomal random hexa‐nucleotide primers (Endoh et al., 2005). The reactions were performed in a SimpliAmp thermocycler (Applied Biosystems) using previously described thermal cycling conditions (Ajamma et al., 2018). The 10‐µl reaction mixture for the multiplex PCR‐HRM contained 1 µl cDNA template, 5 μl of 2× MyTaq HS Mix (Bioline) and 1 μl of 50 μM SYTO‐9 (Life Technologies). Multiplex PCR‐HRM reactions were performed in a Rotor‐Gene Q real‐time PCR thermocycler (Qiagen) using touchdown thermal cycling conditions described in detail elsewhere (Villinger et al., 2017). Each run included cDNA of the sindbis virus as a positive control and no‐template extraction controls and molecular grade water as PCR negative controls. HRM profiles were visualized with Rotor‐Gene Q Series software 2.1.0. All positive samples were separately rerun using primer mixes for each of alphaviruses, flaviviruses and nairoviruses and the same conditions for the multiplex PCR‐HRM runs (Villinger et al., 2017) (Table S1). Amplicons from singleplex runs were purified with an Exo 1‐rSAP combination (Biolabs) and submitted for bidirectional sequencing to Macrogen. Larger fragments using a conventional PCR assay that targets the *Nairovirus* L‐polymerase gene (Table S1) were also amplified, purified and sequenced as previously described (Honig et al., 2004).

#### 2.6.2 Detection of bacterial and protozoan pathogens

Tick, louse, flea and livestock blood samples were also screened for bacteria and protozoa using a combination of PCR‐HRM and conventional PCR. Previously developed primers that target the 16S rRNA gene of *Anaplasma* (Mwamuye et al., 2017), *Ehrlichia* (Mwamuye et al., 2017) and *Rickettsia* (Nijhof et al., 2007), as well as primers that target the 18S ribosomal gene of *Theileria* and *Babesia* parasites (Georges et al., 2001), were used for initial screening (Table S1). Ten‐microlitre reactions that consisted of 2 µl template, 2 µl 5× HOT FIREPol^®^ EvaGreen HRM Mix (Solis BioDyne) and 0.5 µl of each primer at 10 µM concentrations. Cycling was carried out in a Rotor‐Gene Q real‐time PCR thermocycler (Qiagen) as described before (Mwamuye et al., 2017). Positive controls for *Anaplasma* (*A. marginale*) and *Rickettsia* (*R. africae*) (previously detected in *icipe*'s ML‐EID lab from *Amblyomma* spp. ticks) were included in the runs. Resultant HRM profiles were visually inspected with Rotor‐Gene Q Series software 2.1.0, and representative amplicons with unique HRM profiles were purified using an Exo 1‐rSAP combination (Biolabs) and sequenced at Macrogen.

Positive *Ehrlichia* and *Anaplasma* samples were further amplified with a semi‐nested PCR to generate a longer fragment of the 16S rRNA gene (1,030 bp) by combining the Anaplasmataceae‐specific forward primer, EHR16SD (Parola et al., 2001) with universal reverse primers pH1522 (Edwards et al., 1989) and pH1492 (Reysenbach et al., 1992) for first and second round amplification, respectively (Table S1). Primary amplifications were performed using a hot‐start activation step of 95°C for 15 min followed by 1 cycle of 95°C for 20 s, 63°C for 30 s, and 72°C for 90 s, 2 cycles of 95°C for 20 s, 62°C for 30 s, and 72°C for 90 s, 2 cycles of 95°C for 20 s, 61°C for 30 s and 72°C for 90 s, followed with 35 cycles of 95°C for 20 s, 60°C for 30 s and 72°C for 80 s, and a final extension at 72°C for 10 min. The secondary 20‐µl amplification reactions utilized 2 µl of PCR products from primary reactions as templates. The cycling profile consisted of: 95°C for 15 min; 3 cycles of 95°C for 20 s, 61°C for 30 s, and 72°C for 90 s; 37 cycles of 95°C for 20 s, 60°C for 30 s and 72°C for 80 s, and a final extension at 72°C for 10 min. To minimize the risk of contamination, we set up the second reaction in a PCR enclosure and opened only one tube at a time. Products were visualized after gel electrophoresis to confirm the presence of the expected product at 1,030 bp. For *Rickettsia*, all samples with positive HRM profiles were further amplified with Rick‐*ompB* primers (Roux & Raoult, 2000) targeting a 856‐bp region of the outer membrane protein B gene of all *Rickettsia* species (Table S1). Positive samples were prepared for sequencing using the QuickClean II Gel Extraction Kit (GenScript) and submitted to Macrogen for bidirectional sequencing.

### 2.7 Phylogenetic analysis

All sequences were edited and aligned using Geneious alignment in Geneious Prime version 2019.0.4 software (Biomatters). Homologous sequences of reference and sequence entries closely related with each of the individual sequences generated in this study were identified through BLAST nucleotide searches against the GenBank nr database (Altschul et al., 1990). Each of the datasets compiled in this manner were aligned, and the terminal regions corresponding to the primer sequences were removed prior to phylogenetic analysis. Maximum likelihood phylogenies were inferred for each gene using PhyML version 3.0., employing the Akaike information criterion for automatic selection for appropriate model of evolution (Guindon et al., 2010). Trees were visualized and edited in Figtree 1.4 (Rambaut, 2014).

### 2.8 Estimation of individual‐level pathogen prevalences from pooled samples

Individual‐level prevalences of pathogens detected in pooled samples were estimated by a maximum likelihood approach in a frequentist model. True prevalence estimates within vector populations assumed 100% sensitivity and specificity of pooled‐sample results and took into account the number of individuals in each pool tested (Cowling et al., 1999; Williams & Moffitt, 2001). The computations were performed online using Epitools an epidemiological calculator accessed from https://epitools.ausvet.com.au/ppvariablepoolsize (Sergeant, 2018).

## 3 RESULTS

### 3.1 Vectors sampled

A total of 456 ticks (434 adults and 22 nymphs), 28 lice (*Haematopinus suis*) and two fleas (*Ct. felis*) collected from cattle, goats, sheep and pigs at LMs and SHs were analysed in this study. Over 80% of the vectors collected at LMs and SHs came from cattle (Table S2). This was partially due to the fact that 60% of the animals sampled at each of these locations were cattle, which were generally more tick‐infested than goats, sheep or pigs. The lice were primarily collected from pigs at SHs, and the fleas were collected from cattle.

Representative specimens of *Rhipicephalus evertsi* (one adult), *Rhipicephalus appendiculatus* (one adult, one nymph), *Amblyomma gemma* (one adult), *Amblyomma variegatum* (one adult, one nymph), *Haemaphysalis* sp. (one adult), *Rhipicephalus decoloratus* (one adult) and *Rhipicephalus* sp. (six adults, one nymph), identified morphologically (Figure S1), were selected for molecular tick identification (Table 1). Molecular identifications concurred with morphological identifications for *Rh. appendiculatus* (T16), *Rh. decoloratus* (T134) and *Am. variegatum* (T199). However, we resolved a tick specimen (T105) that we morphologically identified as *Rh. decoloratus* to be *Rhipicephalus microplus* based on its 16S rRNA sequence homology. The ITS2 sequence of an *Am. gemma* (T222) had highest homology with *Amblyomma hebraeum*, as there was no other *Am. gemma* ITS2 reference in the GenBank database. Seven out of nine specimens of *Rhipicephalus*, *Haemaphysalis* and *Amblyomma* spp. that could not be identified to species level by morphology alone were identified based on sequence homologies of at least two of the markers. The most commonly sampled tick species were *Rh. decoloratus* (35%) and *Am. variegatum* (30%).

**TABLE 1:** Comparison of molecular and morphological identification of ticks

Sample identification	Morphological identification	16S rRNA (% homology, GenBank accession)	ITS2 (% homology, GenBank accession)	CO1 (% homology, GenBank accession)	Consensus identification (GenBank accessions)
T15	*Rhipicephalus* sp.	*Rh. decoloratus* (100, EU918193)	*Boophilus decoloratus* (96.7, U97716)	–	*Rh. decoloratus* (16S: MN266914; ITS2: MN266918)
T16	*Rh. appendiculatus*	*Rh. appendiculatus* (99.35, KC503257)	*Rh. appendiculatus* (99.8, KX276951)	*Rh. appendiculatus* (100, KC503257)	*Rh. appendiculatus* (16S: MN266911; ITS2: MN266949; CO1: MN294736)
T34	*Rhipicephalus* sp.	*Rh. microplus* (99.2, MH513311)	*Rh. microplus* (99.6, KC503265)	*Rh. microplus* (100, KY678120)	*Rh. microplus* (16S: MN264523; ITS2: MN266952; CO1: MN294738)
T50	*Rhipicephalus* sp.	*Rh. microplus* (99.3, KY020993)	*Rh. microplus* (99.6, MG721035)	*Rh. microplus* (100, KY678120)	*Rh. microplus* (16S: MN264524; ITS2: MN266953; CO1: MN294739)
T62	*Rhipicephalus* sp.	*Rh. decoloratus* (100, EU918193)	*Boophilus decoloratus* (96.7, U97716)	–	*Rh. decoloratus* (16S: MN266915; ITS2: MN266919)
T63	*Rhipicephalus* sp. nymph	*Rh. appendiculatus* (99.35, KC503257)	*Rh. appendiculatus* (99.35, KC503257)	*Rh. appendiculatus* (99.9, KC503257)	*Rh. appendiculatus* (16S: MN266912; ITS2: MN266950; CO1: MN294737)
T105	*Rh. decoloratus*	*Rh. microplus* (99.1, MH513311)	–	–	*Rh. microplus* (16S: MN264525)
T134	*Rh. decoloratus*	*Rh. decoloratus* (100, EU918193)	*Boophilus decoloratus* (96.7, U97716)	–	*Rh. decoloratus* (16S: MN266916; ITS2: MN266921)
T192	*Haemaphysalis* sp.	*Ha. elliptica* (95.6, HM068961)	*Ha. erinacei* (88, KU364288)	*Ha. erinacei* (99.3, KU880573)	*Haemaphysalis* sp. (16S: MN264214; ITS2: MN266944;CO1: MN294735)
T199	*Am. variegatum*	*Am. variegatum* (99.3, L34312)	*Am. variegatum* (100, HQ856803)	–	*Am. variegatum* (16S: MN266929; ITS2: MN401349)
T218	*Rhipicephalus* sp. nymph	*Rh. appendiculatus* (99.51, KC503257)	*Rh. appendiculatus* (99.73, KY457500)	–	*Rh. appendiculatus* (16S: MN266913; ITS2: MN266951)
T222	*Am. gemma*	–	*Am. hebraeum* (99.65, KY457490)	–	*Am. gemma* (ITS2: MN401350)
T311	*Amblyomma* sp. nymph	*Am. variegatum* (99.3, L34312)	*Am. variegatum* (100, HQ856803)	–	*Am. variegatum* (16S: MN266930; ITS2: MN401351)
T321	*Rhipicephalus* sp.	*Rh. simus* (96.28, KJ613641)	–	–	*Rhipicephalus* sp. (16S: MN266945)

### 3.2 Pathogens detected

**FIGURE 2:**
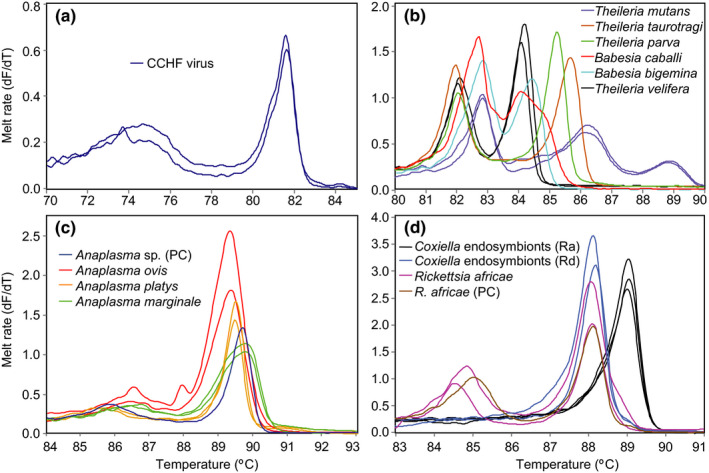
Melt rate profiles. (a) CCHF virus RdRp amplicons, (b) *Theileria/Babesia* 18S rRNA amplicons, (c) *Anaplasma* 16SrRNA amplicons and (d) *Rickettsia/Coxiella* 16S rRNA amplicons. PC, positive control; Ra, *Rh. appendiculatus*; Rd, *Rh. decoloratus* [Colour figure can be viewed at wileyonlinelibrary.com]

**FIGURE 3:**
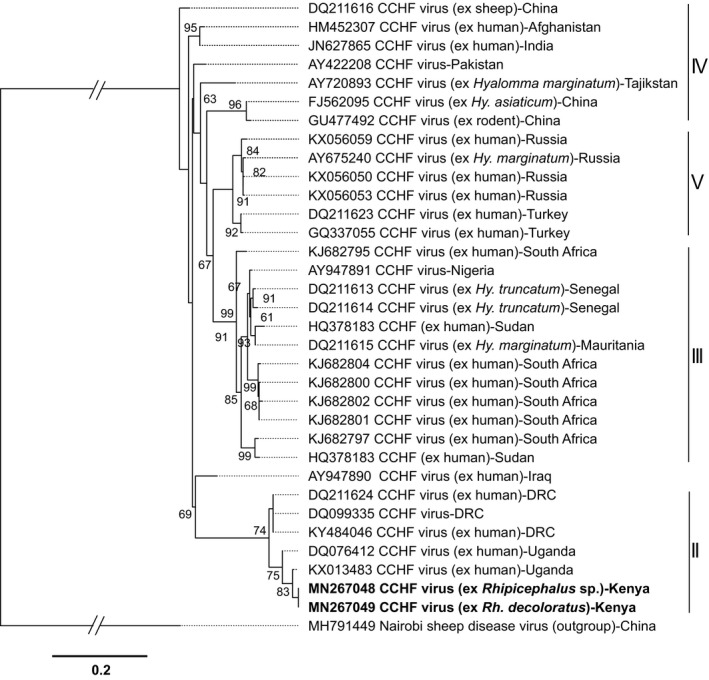
Maximum likelihood phylogeny of Crimean‐Congo haemorrhagic fever virus strains inferred from 34 aligned 434‐nt segments of the L‐segment (RdRp gene). GenBank accession numbers and country of origin are indicated for each sequence. Accession numbers for sequences from this from this study are in bold. Isolation sources in applicable sequences are also highlighted. Bootstrap values at the major nodes are of percentage agreement among 1,000 replicates. The branch length scale represents substitutions per site. The gaps indicated in the branches to the Nairobi sheep disease out‐group represent 0.8 substitutions per site. The sequences from this study fall into African genotype II as indicated by the vertical bars

We detected *Anaplasma* and *Rickettsia* bacteria, *Babesia*, *Theileria*, *Hepatozoon* protozoa and CCHF virus (Figure 2) in ticks and lice collected from 13 LMs and 13 SHs across the three sampled counties (Table 2). Out of the 333 pools tested (Table S2), one *Rh. decoloratus* and one *Rhipicephalus* sp. were positive for CCHF virus (deposited GenBank accessions MN267048, MN267049) (0.62% estimated true prevalence). These ticks were removed from cattle at two SHs. The CCHF virus isolates identified fall into the genotype II clade, which includes isolates from Uganda and the Democratic Republic of Congo (DRC) (Figure 3). Their nucleotide sequence identity was highest (98.6%) to the Nakiwogo (GenBank accession KX013483) strain isolated from Uganda (Simpson et al., 1967).

**TABLE 2:** Vector‐borne pathogens detected in pools of ticks and lice from livestock markets and slaughterhouses

Pathogen	*Rhipicephalus* spp.	*Rh. decoloratus*	*Rh. appendiculatus*	*Rh. evertsi*	*Rhipicephalus* sp.	*Amblyomma* spp.	*Am. variegatum*	*Am. gemma*	*H. suis*	Total
Total pools	215^a^	108	33	18	54	99	96	3	17	333^a^
*A. marginale*	6 (1.88%)	4 (2.44%)^b^	–	–	2 (2.90%)	–	–	–	–	6 (1.24%)
*A. ovis*	6 (1.88%)	2 (1.21%)	–	1 (4.17%)	3 (4.35%)	–	–	–	–	6 (1.24%)
*A. platys*	10 (3.15%)	5 (3.05%)	3 (5.51%)	–	2 (2.92%)	–	–	–	–	10 (2.07%)
*B. bigemina*	2 (0.63%)	1 (0.61%)	1 (1.84%)	–	–	1 (0.75%)	1 (0.77%)	–	–	3 (0.62%)
*B. caballi*	–	–	–	–	–	8 (6.14%)	8 (6.28%)	–	–	8 (1.66%)
*H. canis*	1 (0.31%)	1 (0.61%)	–	–	–	–	–	–	–	1 (0.21%)
*R. africae*	8 (2.52%)	3 (1.83%)	1 (1.82%)	–	4 (5.89%)	83 (77.45%)	82 (78.95%)	1 (33.33%)	1 (3.71%)	92 (19.85%)
*T. mutans*	18 (5.64%)	12 (7.32%)	–	–	6 (8.83%)	1 (0.75%)	1 (0.76%)	–	–	19 (3.93%)
*T. parva*	1 (0.31%)	–	–	–	1 (1.45%)	–	–	–	–	1 (0.21%)
*T. taurotragi*	6 (1.88%)	2 (1.21%)	1 (1.80%)	–	3 (4.38%)	–	–	–	–	6 (1.24%)
*T. velifera*	1 (0.31%)	–	–	–	1 (1.45%)	2 (1.49%)	2 (1.53%)	–	–	3 (0.62%)
CCHF virus	2 (0.62%)	1 (0.61%)	–	–	1 (1.45%)	–	–	–	–	2 (0.41%)

^a^ These totals also include *Rh. microplus*, *Haemaphysalis* sp. and *Ct. felis* pools that were not positive for any pathogens.

^b^ Estimated individual‐level prevalence percentages (in brackets) were calculated based on the size of each pool tested.

Eighty‐two out of 96 pools of *Am. variegatum,* three pools of *Rh. decoloratus*, four pools of *Rhipicephalus* sp., one pool of *Rh. appendiculatus*, one pool of *Am. gemma* and one pool of *H. suis* were positive for *R. africae* (deposited GenBank accessions MN294740–MN294749) (Table 2). These *R. africae*‐positive ectoparasites were removed from cattle, sheep, goats and pigs. Two of the *R. africae* sequences from this study were identical to those previously detected in *Am. variegatum* ticks in Asembo in Kenya (GenBank accession KF660534) and another to a strain detected in a patient diagnosed with African tick bite fever in Tanzania (unpublished; GenBank accession KU721071). *Rickettsia africae* variants in this study were characterized by base substitutions in several positions and possessed a four‐base insertion that is absent from most Kenyan isolates (Figure S2).

We detected *A. platys* (deposited GenBank accessions MN266939–MN266941) in five pools of *Rh. decoloratus*, two pools of *Rhipicephalus* sp. and three pools of *Rh. appendiculatus*, all obtained from cattle (Table S3). *Anaplasma marginale* (deposited GenBank accessions MN266931–MN266935) was detected in four pools of *Rh. decoloratus* and two pools of *Rhipicephalus* sp. *Anaplasma ovis* (deposited GenBank accessions MN266936–MN266938) was detected in two pools of *Rh. decoloratus*, three pools of *Rhipicephalus* sp. and one pool of *Rh. evertsi* from goats and cattle.

**FIGURE 4:**
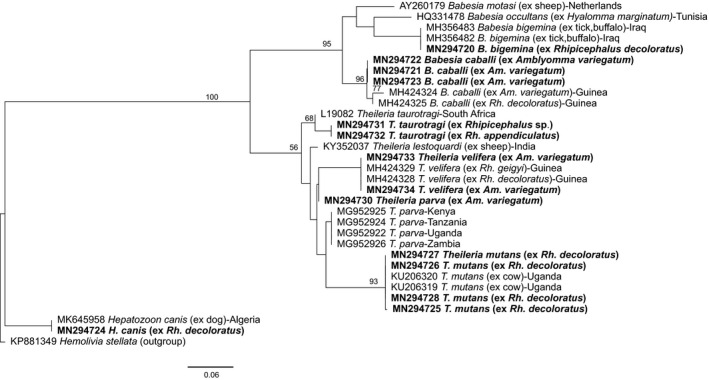
Maximum likelihood phylogeny of apicomplexan protozoa inferred from 32 aligned 502‐nt segments of the 18S rRNA gene. GenBank accession numbers and isolation sources are indicated for each sequence. Accession numbers for sequences from this study are in bold. Bootstrap values at the major nodes are of percentage agreement among 1,000 replicates. The branch length scale represents substitutions per site

Only one *Rhipicephalus* sp. tick pool was positive for *T. parva* (GenBank accession MN294730) (Table 2). Twelve out of 108 pools of *Rh. decoloratus* were positive for *Theileria mutans* (deposited GenBank accessions MN294725–MN294729), while two pools were positive for *Theileria taurotragi* (deposited GenBank accessions MN294731–MN294732). In *Rhipicephalus* sp., six pools were positive for *T. mutans*, three for *T. taurotragi* and one for *Theileria velifera* (deposited GenBank accessions MN294733–MN294734). *Theileria mutans* was also detected in one *Rh. appendiculatus* and one *A. variegatum* pool. All *Theileria* spp. positive ticks were removed from cattle (Table S3). We detected *Babesia caballi* (deposited GenBank accessions MN294721–MN294723) exclusively in eight *Am. variegatum* tick pools. Single pools each of *Rh. decoloratus*, *Rh. appendiculatus* and *Am. variegatum* were positive for *Babesia bigemina* (deposited GenBank accession MN294720). One pool of *Rh. decoloratus* was positive for *Hepatozoon canis* (deposited GenBank accession MN294724). The phylogenetic relationships of the apicomplexan parasite sequences identified in this study with homologous pathogen sequences are shown in Figure 4.

**FIGURE 5:**
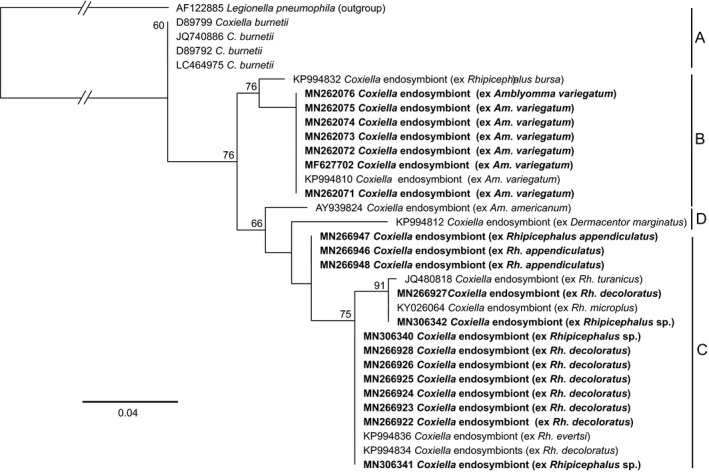
Maximum likelihood phylogeny of tick‐associated *Coxiella* endosymbionts inferred from 33 aligned 279‐nt segments of the 16S rRNA gene. GenBank accession numbers and tick species of origin are indicated for each sequence. Accession numbers for sequences from this study are in bold Bootstrap values at the major nodes are of percentage agreement among 1,000 replicates. The branch length scale represents substitutions per site. The gaps indicated in the branches to the *L. pneumophila* out‐group represent 0.12 substitutions per site. Sequences from this study and those from GenBank fall into three genotypes: A = *Coxiella burnetii*; B = *Coxiella* endosymbionts of *Amblyomma* spp. ticks; C = *Coxiella* endosymbionts of *Rhipicephalus* spp. ticks; D = *Coxiella* endosymbionts of *Dermacentor* and *Amblyomma* spp. ticks

In addition to these pathogens, we detected *Coxiella* endosymbionts (deposited GenBank accessions MN262071–MN262076, MN266922–MN266928, MN266946–MN266948), which are phylogenetically close to, but distinct from, *Coxiella burnetii*, the pathogen responsible for Q fever, in all the genera of ticks except in *Haemaphysalis*. The *Coxiella* endosymbionts characterized in this study fell into the group B and C clades of previously detected tick *Coxiella* endosymbionts of ticks (Figure 5).

No DNA/RNA of the pathogens evaluated in this study was detected in the flea specimens. All of the 33 selected associated livestock blood samples were negative for *R. africae* and CCHF virus. Thirty‐one of these blood samples were from animals (28 cattle and three pigs) from which *R. africae*‐positive *Am. variegatum* ticks were collected, while the other two were from the cattle from which the two CCHF virus‐positive *Rhipicephalus* spp. were obtained.

## 4 DISCUSSION

### 4.1 CCHF virus detection in ticks

We detected CCHF virus in ticks removed from cattle destined for slaughter at two SHs. This is the first description of CCHF virus in *Rh. decoloratus* ticks in Kenya, with previous studies reporting detection only in hyalommid ticks from the North Eastern region (Sang et al., 2011). This suggests that other tick species besides *Hyalomma* spp. may be supporting the local transmission of the virus. As the infected *Rhipicephalus* spp. ticks in this study were blood‐fed and collected from livestock, we also tested the blood of the livestock from which they came from for CCHF virus, but they were negative. Association between infected ticks and seropositivity is common; however, ticks can also be found on seronegative animals and vice versa (Spengler et al., 2016). Domestic animals, especially sheep, have been shown to be asymptomatic carriers of the virus (Spengler et al., 2016), acting as reservoirs of infection (via ticks) to humans, who suffer significant morbidity (Ergönül, 2006). While *Hyalomma* ticks are the natural vector and reservoir of CCHF virus, other tick genera, such as *Rhipicephalus*, have been found infected with the virus (Nabeth et al., 2004; Fakoorziba et al., 2015; Hoogstraal, 1979). *Rhipicephalus* spp. have also transmitted CCHF virus in laboratory settings and have been implicated in the transmission of CCHF virus (Balinandi et al., 2018; Ergönül, 2006). Therefore, *Rhipicephalus* spp. ticks may support transmission of the virus in areas where *Hyalomma* spp. are absent. However, confirmation of this requires comprehensive competency studies, and an understanding of the landscape epidemiology of this virus and its transmission is in its early stages.

In Africa, there are three distinct clades of CCHF virus and the close phylogenetic relationship between our isolates and the Nakiwogo strain isolated in Uganda is not surprising (Ergönül, 2006; Lukashev et al., 2016) given the geographical proximity of our study site to Uganda and the extensive trade in live animals between the two countries. This finding supports the circulation of a single strain of virus between the two countries, which may be facilitated through cross‐border movement of infected livestock. At‐risk groups for CCHF virus infection include farmers, veterinarians and abattoir and healthcare workers (Cook, De Glanville, et al., 2017; Ergönül, 2006). CCHF outbreaks have not been reported in Kenya since the year 2000 when a fatal case in western Kenya showed the possibility of the virus circulating in the region. However, Lwande et al. (2012) found a 23% human seroprevalence of IgG antibodies to CCHF virus in North Eastern Kenya, and infection has been reported after skin contact with livestock, blood spatters during slaughtering, tick bites and when healthcare workers take care of haemorrhaging patients (Ergönül, 2006). Our findings therefore highlight the potential for human exposure to CCHF virus at these and other LMs and SHs, and at public health facilities, and emphasize the need for routine surveillance for this pathogen and adopting a One Health approach. Other LM/SH‐based surveillance studies in Kenya have described the occurrence of other arboviruses in ticks, which indicates the importance of ticks in their epidemiology (Lwande et al., 2013; Sang et al., 2006, 2011). While most of these studies targeted pastoralist regions, our findings demonstrate that the risk of human exposure to tick‐borne arboviruses is also present in tropical smallholder systems in East Africa.

While *Hyalomma* spp. ticks are the chief vectors of CCHF virus, other species may also be important to transmission ecologies due to cofeeding transmission between infected and non‐infected ticks, even in the absence of viraemia in the host. An infected tick may transmit a virus to a non‐infected cofeeding tick without the host having detectable virus in its blood (Kazimírová et al., 2017). Such non‐viraemic transmission is presumed to contribute to amplification of CCHF virus in nature because the virus can be transmitted among ticks even without detectable viraemia in the host (Bente et al., 2013).

### 4.2 *Rickettsia africae* in ticks and lice

We demonstrated a high prevalence (78.95%; estimated true prevalence) of *R. africae*, the agent of African tick bite fever (ATBF, also known as African tick typhus) in humans, in *Am. variegatum* ticks collected mostly from cattle. Ever since the first description in Kenya of *R. africae* in *Amblyomma* ticks from the Maasai Mara region (Macaluso et al., 2003), high infection rates in *Amblyomma* ticks have been reported at SHs in Mombasa and Nairobi (Mutai et al., 2013), Siaya County, which borders Busia County (Maina et al., 2014), pastoral communities in North Eastern Kenya (Koka et al., 2017), the Shimba Hills National Reserve (Mwamuye et al., 2017), Baringo County (Omondi et al., 2017) and the Maasai Mara National Reserve (Oundo et al., 2020). *Rickettsia africae* has similarly been reported in *Amblyomma* ticks from Cameroon (Ndip, Fokam, et al., 2004), Zimbabwe (Beati et al., 1995), Senegal (Kelly et al., 2010) and the Central African Republic (CAR) (Dupont et al., 1995). We also detected *R. africae* at much lower prevalence in rhipicephaline ticks, and for the first time, we are aware of in *H. suis* lice. However, this novel finding is not surprising as lice are known vectors of other SFG rickettsiae (Hornok et al., 2010), but there is a paucity of studies that have surveyed rickettsiae in lice in Africa. The two fleas, which were negative for pathogen DNA/RNA, may have been an accidental finding on cattle. However, *Ct. felis* is not as host‐restricted as *Ctenocephalides canis* and has been found infesting a wide range of host other than felines (Singh et al., 2011).

Our finding that all 34 livestock blood samples, from which the *R. africae*‐positive ticks were obtained, were negative for the pathogen reinforces the notion that *Amblyomma* ticks are the major reservoir of the pathogen, but also indicates a low transmissibility to livestock. Since these ticks mostly parasitize large ruminants, it is evident that cattle play an important role in the epidemiology of ATBF by providing an abundant blood‐meal source, as described previously for *R. conorii* by Kelly et al. (1991).

In travel medicine, ATBF, which is characterized by headaches, inoculation eschar, rash and myalgia (Jensenius et al., 2003), is believed to be only second to malaria as the cause of febrile illness in travellers to sub‐Saharan Africa (SSA). Most acute cases have been reported in tourists and foreign travellers with some fatal cases (Rutherford et al., 2004). Its seroprevalence is usually high in native populations, but few acute cases have been reported (Kelly et al., 1991; Ndip, Bouyer, et al., 2004). This may be due to exposure at an early age leading to only mild clinical cases that are ignored, poor visibility of inoculation eschars on pigmented skin and lack of diagnostic capacity at most health centres (Jensenius et al., 2003). Alternatively, some *R. africae* may be more virulent than others. In this study, we found *R. africae* variants that have been reported in previous studies (Kimita et al., 2016; Macaluso et al., 2003; Maina et al., 2014). The differences found in the nucleotide composition of the *omp* B gene, which codes for the most immuno‐dominant surface cell antigen of *Rickettsia*, could possibly affect the virulence of *R. africae* variants. Surface cell antigens are involved in cellular adhesion of *Rickettsia* and subsequent entry into cells (Blanc et al., 2003). The hypothesis that variants with an intact *omp* B gene are less virulent than those with the deletion (Maina et al., 2014) may explain the absence of acute ATBF cases in Kenya, despite the high seroprevalence. This is supported by the evidence that genome reduction may lead to increased virulence in *Rickettsia* (Fournier et al., 2009). However, it remains to be seen if some of these variants can be detected in febrile patients in our study area. Clearly, there is a need for studies that focus on the public health aspect of this pathogen in endemic areas.

### 4.3 *Theileria*, *Babesia* and *Anaplasma* spp. in ticks

We detected *A. marginale*, the cause of gall sickness, *B. bigemina,* which causes redwater, and *T. parva*, which causes East Coast fever in 1.88%, 0.63% and 0.31% (estimated true prevalences) of rhipicephaline ticks, respectively. These three diseases are major impediments to livestock production in Kenya and SSA, causing severe loss of production in affected animals (Wesonga et al., 2010; Woolhouse et al., 2015). We recently found *T. parva* more frequently in *Rh. appendiculatus* (15.7% of tick pools) sampled in the Maasai Mara National Reserve, where no *Babesia* was detected (Oundo et al., 2020). The absence of *T. parva* in animal blood samples in this study may be partly explained by its biology, where most of its life cycle is found in the lymphoid system and only multiplies in RBC for completion of its life cycle (Mans et al., 2015). Accordingly, we found higher prevalence in ticks of the mildly pathogenic *Theileria* spp., *T. taurotragi*, *T. velifera* and *T. mutans* than reported by Njiiri et al. (2015) in calves in Busia, Kenya and by Lorusso et al. (2016) in Nigerian cattle. Nonetheless, these species can also cause theileriosis in immuno‐compromised animals. We also detected *A. platys*, the cause of canine cyclic thrombocytopenia, in several pools of *Rhipicephalus* ticks from cattle. This pathogenic bacterium has been reported in other studies in ticks and blood from livestock (Ben Said et al., 2017; Lorusso et al., 2016; Omondi et al., 2017), and recent evidence suggests that *A. platys* may infect humans, posing a risk in cases of opportunistic tick bites (Arraga‐Alvarado et al., 2014; Breitschwerdt et al., 2014; Maggi et al., 2013).

### 4.4 *Coxiella* endosymbionts of ticks

As in recent studies by Mwamuye et al. (2017) and Oundo et al. (2020), we also obtained *Coxiella* endosymbiont sequences from *Rickettsia* 16S rRNA primer amplicons. Previous studies have shown that these endosymbionts, which are closely related to the pathogen responsible for Q fever, *C. burnetii*, provide additional essential nutrients and reproductive fitness to ticks. Their elimination with antibiotic treatment was shown to negatively impact the fitness of the lone star tick *Amblyomma americanum* (Zhong et al., 2007). The phylogenetic codivergence between the different tick species and their *Coxiella* endosymbionts shows the high specificity of these endosymbionts to their tick hosts. Four phylogenetic clades (A–D) have been described for tick‐associated *Coxiella* endosymbionts. The sequences of endosymbionts from this study fell into groups B and C. Group B consists of *Coxiella* endosymbionts of *Amblyomma* and *Ornithodoros*, while group C consists of rhipicephaline endosymbionts (Duron et al., 2015). These endosymbionts are non‐pathogenic. However, it is important to note that there is evidence that *C. burnetii* evolved recently from a maternally inherited symbiont of ticks (Duron et al., 2015).

## 5 CONCLUSIONS

We identified an array of pathogens of both veterinary and public health importance in vectors collected from domestic animals at LMs and SHs. Significantly, the host animals were either being traded to destinations that were different from their origin or taken to slaughter, carrying infected vectors. These findings show how livestock trade can be the driver for new foci of infection in new areas, with risks to livestock from pathogens identified in this study, such as *T. parva*, *A. marginale* and *B. bigemina*. Furthermore, the presence of CCHF virus at SHs exposes abattoir workers, meat inspectors, butchers and consumers to the haemorrhagic disease, which is highly fatal. The high prevalence of *R. africae* detected in *Am. variegatum* ticks shows the high risk of transmission of the pathogen, which causes ATBF in humans in case of a tick bite. The zoonotic pathogens detected here cause febrile illness that can be clinically difficult to differentiate from malaria or other non‐specific fevers. Indeed, a large majority of non‐malarial febrile cases are never properly diagnosed. Therefore, evidence of their possible circulation and risk for human infection warrants their inclusion, if not routinely due to limitations in clinical differential diagnostics, at least in routine prospective surveys in health centres receiving febrile patients.

## ETHICAL APPROVAL

The authors confirm that the ethical policies of the journal, as noted on the journal's author guidelines page, have been adhered to, and the appropriate ethical review committee approval has been received. This study was nested within the Zoonoses in Livestock in Kenya (ZooLinK) project. Tick, louse, flea and blood samples were collected from cattle, goats, sheep and pigs at LMs or presented for slaughter at SHs and approved by the International Livestock Research Institute Institutional Animal Care and Use Committee (ref IACUC‐RC 2017‐04). Data from human owners of livestock were collected after approval by the International Livestock Research Institute (ILRI) Institutional Research Ethics Committee (ref ILRI‐IREC 2017‐08/2). Both committees are licensed by the National Commission for Science, Technology and Innovation (NACOSTI) in Kenya.

## CONFLICT OF INTEREST

The authors declare that they have no competing interests.

## AUTHOR CONTRIBUTIONS

TC, DKM, LCF, EMF and JV designed the study and sampling. TC did the identification and laboratory work. TC and JV analysed the results. TC wrote the original manuscript, while DKM, LCF, ADSB, EMF and JV edited and reviewed the manuscript. All the authors approved the final manuscript.

## Supporting information

Fig S1

Fig S2

Table S1

Table S2

Table S3

## Data Availability

All nucleotide sequence data generated in the study were deposited into the GenBank database under the following accessions: arthropod 16S: MN264214, MN264523–MN264525, MN266911–MN266916, MN266929, MN266930, MN266945; arthropod ITS2: MN266918, MN266919, MN266921, MN266944, MN266949–MN266953, MN401349–MN401351; arthropod CO1: MN294735–MN294739, CCHF: MN267048, MN267049; *Rickettsia* spp.: MN294740–MN294749, MN266939–MN266941; *Anaplasma* spp.: MN266931–MN266941; *Theileria* spp.: MN294725–MN294734; *Babesia* spp.: MN294720–MN294723; *H. canis*: MN294724; *Coxiella* spp.: MN262071–MN262076; MN266922–MN266928, MN266946–MN266948.

## References

[tbed13911-bib-0001] Adjou Moumouni, P. F., Aboge, G. O., Terkawi, M. A., Masatani, T., Cao, S., Kamyingkird, K., Jirapattharasate, C., Zhou, M. O., Wang, G., Liu, M., Iguchi, A., Vudriko, P., Ybanez, A. P., Inokuma, H., Shirafuji‐Umemiya, R., Suzuki, H., & Xuan, X. (2015). Molecular detection and characterization of *Babesia bovis*, *Babesia bigemina*, *Theileria* species and *Anaplasma marginale* isolated from cattle in Kenya. Parasites & Vectors, 8, 496. 10.1186/s13071-015-1106-9 26420543 PMC4589125

[tbed13911-bib-0002] Ajamma, Y. U., Onchuru, T. O., Ouso, D. O., Omondi, D., Masiga, D. K., & Villinger, J. (2018). Vertical transmission of naturally occurring Bunyamwera and insect‐specific flavivirus infections in mosquitoes from islands and mainland shores of Lakes Victoria and Baringo in Kenya. PLoS Neglected Tropical Diseases, 12(11), e0006949. 10.1371/journal.pntd.0006949 30452443 PMC6287884

[tbed13911-bib-0003] Altschul, S. F., Gish, W., Miller, W., Myers, E. W., & Lipman, D. J. (1990). Basic local alignment search tool. Journal of Molecular Biology, 215, 403–410. 10.1016/S0022-2836(05)80360-2 2231712

[tbed13911-bib-0004] Arraga‐alvarado, C. M., Qurollo, B. A., Parra, O. C., Berrueta, M. A., Hegarty, B. C., & Breitschwerdt, E. B. (2014). Case report: Molecular evidence of *Anaplasma platys* infection in two women from Venezuela. American Journal of Tropical Medicine and Hygiene, 91, 1161–1165. 10.4269/ajtmh.14-0372 PMC4257640 25266347

[tbed13911-bib-0005] Baba, M., Masiga, D. K., Sang, R., & Villinger, J. (2016). Has Rift Valley fever virus evolved with increasing severity in human populations in East Africa? Emerging Microbes and Infections, 5(6), e58. 10.1038/emi.2016.57 27329846 PMC4932650

[tbed13911-bib-0006] Balinandi, S., Patel, K., Ojwang, J., Kyondo, J., Mulei, S., Tumusiime, A., Lubwama, B., Nyakarahuka, L., Klena, J. D., Lutwama, J., Strӧher, U., Nichol, S. T., & Shoemaker, T. R. (2018). Investigation of an isolated case of human Crimean‐Congo hemorrhagic fever in Central Uganda. International Journal of Infectious Diseases, 68, 88–93. 10.1016/j.ijid.2018.01.013 29382607 PMC5893389

[tbed13911-bib-0007] Baron, M. D., & Holzer, B. (2015). Nairobi sheep disease virus/Ganjam virus. Scientific and Technical Review of the Office International des Epizooties, 34, 411–417. 10.20506/rst.34.2.2367 26647464

[tbed13911-bib-0008] Beati, L., Kelly, P. J., Matthewman, L. A., Mason, P. R., & Raoult, D. (1995). Prevalence of *Rickettsia*‐like organisms and spotted fever group rickettsiae in ticks (Acari: Ixodidae) from Zimbabwe. Journal of Medical Entomology, 32, 787–792. 10.1093/jmedent/32.6.787 8551500

[tbed13911-bib-0009] Ben Said, M., Belkahia, H., El Mabrouk, N., Saidani, M., Alberti, A., Zobba, R., Cherif, A., Mahjoub, T., Bouattour, A., & Messadi, L. (2017). *Anaplasma platys*‐like strains in ruminants from Tunisia. Infection, Genetics and Evolution, 49, 226–233. 10.1016/j.meegid.2017.01.023 28130168

[tbed13911-bib-0010] Bente, D. A., Forester, N. L., Watts, D. M., Mcauley, A. J., Whitehouse, C. A., & Bray, M. (2013). Crimean‐Congo hemorrhagic fever: History, epidemiology, pathogenesis, clinical syndrome and genetic diversity. Antiviral Research, 100(1), 159–189. 10.1016/j.antiviral.2013.07.006 23906741

[tbed13911-bib-0011] Blanc, G., Ngwamidiba, M., Ogata, H., Fournier, P., Claverie, J., & Raoult, D. (2003). Molecular evolution of *Rickettsia* aurface antigens: Evidence of positive selection. Molecular Biology and Evolution, 22(10), 2073–2083. 10.1093/molbev/msi199 15972845

[tbed13911-bib-0012] Brahma, R. K., Dixit, V., Sangwan, A. K., & Doley, R. (2014). Identification and characterization of *Rhipicephalus* (Boophilus) *microplus* and *Haemaphysalis bispinosa* ticks (Acari: Ixodidae) of northeast India by ITS2 and 16S rDNA sequences and morphological analysis. Experimental & Applied Acarology, 62(2), 253–265. 10.1007/s10493-013-9732-4 23990074

[tbed13911-bib-0013] Breitschwerdt, E. B., Hegarty, B. C., Qurollo, B. A., Saito, T. B., Maggi, R. G., Blanton, L. S., & Bouyer, D. H. (2014). Intravascular persistence of *Anaplasma platys*, *Ehrlichia chaffeensis*, and *Ehrlichia ewingii* DNA in the blood of a dog and two family members. Parasites & Vectors, 7, 298. 10.1186/1756-3305-7-298 24984562 PMC4089936

[tbed13911-bib-0014] Brown, L. D., & Macaluso, K. R. (2016). *Rickettsia felis*, an emerging flea‐borne rickettsiosis. Current Tropical Medicine Reports, 3, 27–39. 10.1007/s40475-016-0070-6 27340613 PMC4870301

[tbed13911-bib-0015] Centers for Disease Control and Prevention (CDC) (2003). Pictorial keys to arthropods, reptiles, birds, and mammals of public health significance. Department of Health and Human Services, Centers for Disease Control and Prevention of the U.S. Public Health Service. https://stacks.cdc.gov/view/cdc/13428.

[tbed13911-bib-0016] Chitimia, L., Lin, R. Q., Cosoroaba, I., Braila, P., Song, H. Q., & Zhu, X. Q. (2009). Molecular characterization of hard and soft ticks from Romania by sequences of the internal transcribed spacers of ribosomal DNA. Parasitology Research, 105(4), 907–911. 10.1007/s00436-009-1474-1 19462182

[tbed13911-bib-0017] Cook, E. A. J., De Glanville, W. A., Thomas, L. F., Kariuki, S., Bronsvoort, B. M. C., & Fèvre, E. M. (2017). Working conditions and public health risks in slaughterhouses in western Kenya. BMC Public Health, 17(1), 14. 10.1186/s12889-016-3923-y 28056885 PMC5217581

[tbed13911-bib-0018] Cook, E. A. J., Grossi‐Soyster, E. N., de Glanville, W. A., Thomas, L. F., Kariuki, S., Bronsvoort, B. M. D. C., Wamae, C. N., LaBeaud, A. D., & Fèvre, E. M. (2017). The sero‐epidemiology of Rift Valley fever in people in the Lake Victoria Basin of western Kenya. PLoS Neglected Tropical Diseases, 11(7), e0005731. 10.1371/journal.pntd.0005731 28686589 PMC5517073

[tbed13911-bib-0019] Cowling, D. W., Gardner, I. A., & Johnson, W. O. (1999). Comparison of methods for estimation of individual‐level prevalence based on pooled samples. Preventive Veterinary Medicine, 39(3), 211–225. 10.1016/s0167-5877(98)00131-7 10327439

[tbed13911-bib-0020] de la Fuente, J., Kocan, K. M., Almazán, C., & Blouin, E. F. (2008). Targeting the tick‐pathogen interface for novel control strategies. Frontiers in Bioscience, 1, 6947–6956. 10.1093/cid/cir155 18508707

[tbed13911-bib-0021] Doudier, B., Olano, J., Parola, P., & Brouqui, P. (2010). Factors contributing to emergence of *Ehrlichia* and *Anaplasma spp*. as human pathogens. Veterinary Parasitology, 167, 149–154. 10.1016/j.vetpar.2009.09.016 19836890

[tbed13911-bib-0022] Dunster, L., Dunster, M., Ofula, V., Beti, D., Kazooba‐Voskamp, F., Burt, F., Swanepoel, R., & DeCock, K. M. (2002). First documentation of human Crimean‐Congo hemorrhagic fever. Kenya. Emerging Infectious Diseases, 8(9), 1005–1006. 10.3201/eid0809.010510 12194785 PMC2732535

[tbed13911-bib-0023] Dupont, H. T., Brouqui, P., Faugere, B., & Raoult, D. (1995). Prevalence of antibodies to *Coxiella burnetii*, *Rickettsia conorii*, and *Rickettsia typhi* in seven African countries. Clinical Infectious Diseases, 21, 1126–1133. 10.1093/clinids/21.5.1126 8589132

[tbed13911-bib-0024] Duron, O., Noël, V., McCoy, K. D., Bonazzi, M., Sidi‐Boumedine, K., Morel, O., Vavre, F., Zenner, L., Jourdain, E., Durand, P., Arnathau, C., Renaud, F., Trape, J.‐F., Biguezoton, A. S., Cremaschi, J., Dietrich, M., Léger, E., Appelgren, A., Dupraz, M., … Chevillon, C. (2015). The recent evolution of a maternally‐inherited endosymbiont of ticks led to the emergence of the Q fever pathogen, *Coxiella burnetii* . PLoS Path, 11, e1004892. 10.1371/journal.ppat.1004892 PMC4433120 25978383

[tbed13911-bib-0025] Edwards, E., Rogall, T., Blocker, H., Emde, M., & Bottger, E. C. (1989). Isolation and direct complete nucleotide determination of entire genes. Characterization of a gene coding for 16S ribosomal RNA. Nucleic Acids Research, 17, 7843–7853. 10.1093/nar/17.19.7843 2798131 PMC334891

[tbed13911-bib-0026] Endoh, D., Mizutani, T., Kirisawa, R., Maki, Y., Saito, H., Kon, Y., Morikawa, S., & Hayashi, M. (2005). Species‐independent detection of RNA virus by representational difference analysis using non‐ribosomal hexanucleotides for reverse transcription. Nucleic Acids Research, 33(6), e65. 10.1093/nar/gni064 15817564 PMC1074749

[tbed13911-bib-0027] Ergönül, Ö. (2006). Crimean‐Congo haemorrhagic fever. Lancet Infectious Diseases, 6, 203–214. 10.1016/S1473-3099(06)70435-2 16554245 PMC7185836

[tbed13911-bib-0099] Estrada‐Peña, A., Bouattour, A., Camicas, J.‐L., Walker, A. R. (2004). Ticks of domestic animals in the Mediterranean region: A guide to identification of species. University of Zaragoza.

[tbed13911-bib-0028] Fakoorziba, M. R., Naddaf‐Sani, A. A., Moemenbellah‐Fard, M. D., Azizi, K., Ahmadnia, S., & Chinikar, S. (2015). First phylogenetic analysis of a Crimean‐Congo hemorrhagic fever virus genome in naturally infected *Rhipicephalus appendiculatus* ticks (Acari: Ixodidae). Archives of Virology, 160(5), 1197–1209. 10.1007/s00705-015-2379-1 25742932

[tbed13911-bib-0029] Falzon, L. C., Alumasa, L., Amanya, F., Kang'ethe, E., Kariuki, S., Momanyi, K., Muinde, P., Murungi, M. K., Njoroge, S. M., Ogendo, A., Ogola, J., Rushton, J., Woolhouse, M. E. J., & Fèvre, E. M. (2019). One Health in action: Operational aspects of an integrated surveillance system for zoonoses in western Kenya. Frontiers in Veterinary Science, 6, 252. 10.3389/fvets.2019.00252 31417918 PMC6684786

[tbed13911-bib-0030] Fèvre, E. M., Bronsvoort, B. M. D. C., Hamilton, K. A., & Cleaveland, S. (2006). Animal movements and the spread of infectious diseases. Trends in Microbiology, 14(3), 125–131. 10.1016/j.tim.2006.01.004 16460942 PMC7119069

[tbed13911-bib-0106] Fèvre, E. M., de Glanville, W. A., Thomas, L. F., Cook, E. A. J., Kariuki, S., & Wamae, C. N. (2017). An integrated study of human and animal infectious disease in the Lake Victoria crescent small‐holder crop‐livestock production system, Kenya. BMC Infectious Diseases, 17(1), 457. 28666412 10.1186/s12879-017-2559-6 PMC5493856

[tbed13911-bib-0031] Fèvre, E. M., Picozzi, K., Fyfe, J., Waiswa, C., Odiit, M., Coleman, P. G., & Welburn, S. C. (2005). A burgeoning epidemic of sleeping sickness in Uganda. Lancet, 366(9487), 745–747. 10.1016/S0140-6736(05)67179-6 16125592

[tbed13911-bib-0032] Fournier, P.‐E., El Karkouri, K., Leroy, Q., Robert, C., Giumelli, B., Renesto, P., Socolovschi, C., Parola, P., Audic, S., & Raoult, D. (2009). Analysis of the *Rickettsia africae* genome reveals that virulence acquisition in Rickettsia species may be explained by genome reduction. BMC Genomics, 10, 166. 10.1186/1471-2164-10-166 19379498 PMC2694212

[tbed13911-bib-0033] Fournier, P., Roux, V., Caumes, E., Donzel, M., & Raoult, D. (2017). Outbreak of *Rickettsia africae* infections in participants of an adventure race in South Africa. Clinical Infectious Diseases, 27, 316–323. 10.1086/514664 9709882

[tbed13911-bib-0034] Gachohi, J., Skilton, R., Hansen, F., Ngumi, P., & Kitala, P. (2012). Epidemiology of East Coast fever (*Theileria parva* infection) in Kenya: Past, present and the future. Parasites & Vectors, 5, 194. 10.1186/1756-3305-5-194 22958352 PMC3465218

[tbed13911-bib-0035] Georges, K., Loria, G. R., Riili, S., Greco, A., Caracappa, S., Jongejan, F., & Sparagano, O. (2001). Detection of haemoparasites in cattle by reverse line blot hybridisation with a note on the distribution of ticks in Sicily. Veterinary Parasitology, 99, 273–286. 10.1016/s0304-4017(01)00488-5 11511414

[tbed13911-bib-0036] Guindon, S., Dufayard, J. F., Lefort, V., Anisimova, M., Hordijk, W., & Gascuel, O. (2010). New algorithms and methods to estimate maximum‐likelihood phylogenies: Assessing the performance of PhyML 3.0. Systematic Biology, 59, 307–321. 10.1093/sysbio/syq010 20525638

[tbed13911-bib-0037] Hebert, P., Penton, E. H., Burns, J. M., Janzen, D., & Hallwachs, W. (2004). Ten species in one: DNA barcoding reveals cryptic species in the neotropical skipper butterfly *Astraptes fulgerator* . Proceedings of the National Academy of Sciences of the United States of America, 101(41), 14812–14817. 10.1073/pnas.0406166101 15465915 PMC522015

[tbed13911-bib-0038] Honig, J. E., Osborne, J. C., & Nichol, S. T. (2004). The high genetic variation of viruses of the genus *Nairovirus* reflects the diversity of their predominant tick hosts. Virology, 318, 10–16. 10.1016/j.virol.2003.09.021 14972529

[tbed13911-bib-0039] Hoogstraal, H. (1979). The epidemiology of tick‐borne Crimean‐Congo Hemorrhagic fever in Asia, Europe, and Africa. Journal of Medical Entomology, 15(4), 307–417. 113533 10.1093/jmedent/15.4.307

[tbed13911-bib-0040] Hornok, S., Hofmann‐Lehmann, R., de Mera, I. G., Meli, M. L., Elek, V., Hajtós, I., Répási, A., Gönczi, E., Tánczos, B., Farkas, R., Lutz, H., & de la Fuente, J. (2010). Survey on blood‐sucking lice (Phthiraptera: Anoplura) of ruminants and pigs with molecular detection of *Anaplasma* and *Rickettsia* spp. Veterinary Parasitology, 174(3–4), 355–358. 10.1016/j.vetpar.2010.09.003 20943320

[tbed13911-bib-0041] Inziani, M., Adungo, F., Awando, J., Kihoro, R., Inoue, S., Morita, K., Obimbo, E., Onyango, F., & Mwau, M. (2020). Seroprevalence of yellow fever, dengue, West Nile and chikungunya viruses in children in Teso South Sub‐County, Western Kenya. International Journal of Infectious Diseases, 91, 104–110. 10.1016/j.ijid.2019.11.004 31712089

[tbed13911-bib-0042] Jensenius, M., Fournier, P., Kelly, P., Myrvang, B., & Raoult, D. (2003). African tick bite fever. The Lancet, 3, 557–564. 10.1016/s1473-3099(03)00739-4 12954562

[tbed13911-bib-0043] Jensenius, M., Fournier, P., & Raoult, D. (2017). Rickettsioses and the international traveler. Clinical Infectious Diseases, 39, 1493–1499. 10.1016/j.ijid.2003.06.004 15546086

[tbed13911-bib-0044] Jiang, J., Maina, A. N., Knobel, D. L., Cleaveland, S., Laudisoit, A., Wamburu, K., Ogola, E., Parola, P., Breiman, R. F., Kariuki Njenga, M. & Richards, A. L. (2013). Molecular detection of *Rickettsia felis* and *Candidatus* Rickettsia asemboensis in fleas from human habitats, Asembo, Kenya. Vector Borne and Zoonotic Diseases, 13(8), 550–558. 10.1089/vbz.2012.1123 23675818 PMC3741420

[tbed13911-bib-0045] Kazimírová, M., Thangamani, S., Bartíková, P., Hermance, M., Holíková, V., Štibrániová, I., & Nuttall, P. A. (2017). Tick‐borne viruses and biological processes at the tick‐host‐virus interface. Frontiers in Cellular and Infection Microbiology, 73, 39. 10.3389/fcimb.2017.00339 PMC5526847 28798904

[tbed13911-bib-0046] Kelly, P. J., Lucas, H., Eremeeva, M. E., Dirks, K. G., Rolain, J. M., Yowell, C., & Raoult, D. (2010). *Rickettsia africae*, Western Africa. Emerging Infectious Diseases, 16(3), 571–573. 10.3201/eid1603.090346 20202453 PMC3322006

[tbed13911-bib-0047] Kelly, P. J., Raoult, J. D., & Mason, P. R. (1991). Isolation of spotted fever group *Rickettsia* from triturated ticks using a modification of the centrifugation‐shell vial technique. Transactions of the Royal Society of Tropical Medicine and Hygiene, 85, 397–398. 10.1016/0035-9203(91)90303-g 1949146

[tbed13911-bib-0048] Kilpatrick, A. M., & Randolph, S. E. (2012). Drivers, dynamics, and control of emerging vector‐borne zoonotic diseases. The Lancet, 380, 1946–1955. 10.1016/S0140-6736(12)61151-9 PMC3739480 23200503

[tbed13911-bib-0049] Kimita, G., Mutai, B., Nyanjom, S. G., Wamunyokoli, F., & Waitumbi, J. (2016). Phylogenetic variants of *Rickettsia africae*, and incidental identification of “*Candidatus* Rickettsia moyalensis” in Kenya. PLoS Neglected Tropical Diseases, 10, e0004788. 10.1371/journal.pntd.0004788 27387337 PMC4936727

[tbed13911-bib-0050] Koka, H., Sang, R., Kutima, H. L., Musila, L., & Macaluso, K. (2017). The detection of spotted fever group *Rickettsia* DNA in tick samples from pastoral communities in Kenya. Journal of Medical Entomology, 54, 774–780. 10.1093/jme/tjw238 28073909 PMC5850802

[tbed13911-bib-0051] Kumsa, B., Socolovschi, C., Raoult, D., & Parola, P. (2015). Spotted fever group rickettsiae in ixodid ticks in Oromia, Ethiopia. Ticks and Tick‐Borne Diseases, 6(1), 8–15. 10.1016/j.ttbdis.2014.08.001 25262832

[tbed13911-bib-0052] Latif, A. A., Rowlands, G. J., Punyua, D. K., Hassan, S. M., & Capstick, P. B. (1995). An epidemiological study of tick‐borne diseases and their effects on productivity of zebu cattle under traditional management on Rusinga Island, western Kenya. Preventive Veterinary Medicine, 22, 169–181. 10.1016/0167-5877(94)00408-B

[tbed13911-bib-0053] Lorusso, V., Wijnveld, M., Majekodunmi, A. O., Dongkum, C., Fajinmi, A., Dogo, A. G., Thrusfield, M., Mugenyi, A., Vaumourin, E., Igweh, A. C., Jongejan, F., Welburn, S. C., & Picozzi, K. (2016). Tick‐borne pathogens of zoonotic and veterinary importance in Nigerian cattle. Parasites & Vectors, 9, 217. 10.1186/s13071-016-1504-7 27090756 PMC4836144

[tbed13911-bib-0054] Luce‐fedrow, A., Maina, A. N., Otiang, E., Ade, F., Omulo, S., & Ogola, E. (2015). Isolation of *Candidatus* Rickettsia asemboensis from *Ctenocephalides* fleas. International Journal of Systematic and Evolutionary Microbiology, 66, 4512–4517. 10.1089/vbz.2014.1744

[tbed13911-bib-0055] Lukashev, A. N., Klimentov, A. S., Smirnova, S. E., Dzagurova, K., Drexler, J. F., & Gmyl, A. P. (2016). Phylogeography of Crimean Congo Hemorrhagic fever virus. PLoS One, 11(11), e0166744. 10.1371/journal.pone.0166744 27880794 PMC5120814

[tbed13911-bib-0056] Lutomiah, J., Musila, L., Makio, A., Ochieng, C., Koka, H., Chepkorir, E., Mutisya, J., Mulwa, F., Khamadi, S., Miller, B. R., Bast, J., Schnabel, D., Wurapa, E. K., & Sang, R. (2014). Ticks and tick‐borne viruses from livestock hosts in arid and semiarid regions of the eastern and northeastern parts of Kenya. Journal of Medical Entomology, 51, 269–277. 10.1603/ME13039 24605478

[tbed13911-bib-0057] Lwande, O. W., Irura, Z., Tigoi, C., Chepkorir, E., Orindi, B., Musila, L., Venter, M., Fischer, A., & Sang, R. (2012). Seroprevalence of Crimean Congo hemorrhagic fever virus in Ijara District, Kenya. Vector‐Borne and Zoonotic Diseases, 12, 727–732. 10.1089/vbz.2011.0914 22925021 PMC3438825

[tbed13911-bib-0058] Lwande, O. W., Lutomiah, J., Obanda, V., Gakuya, F., Mutisya, J., Mulwa, F., Michuki, G., Chepkorir, E., Fischer, A., Venter, M., & Sang, R. (2013). Isolation of tick and mosquito‐borne arboviruses from ticks sampled from livestock and wild animal hosts in Ijara District, Kenya. Vector‐Borne and Zoonotic Diseases, 13, 637–642. 10.1089/vbz.2012.1190 23805790 PMC3777299

[tbed13911-bib-0059] Macaluso, K. R., Davis, J. O. N., Alam, U., Korman, A. M. Y., Rutherford, J. S., Rosenberg, R., & Azad, A. F. (2003). Spotted fever group rickettsiae in ticks from the Masai Mara region of Kenya. American Journal of Tropical Medicine and Hygiene, 68, 551–553. 10.4269/ajtmh.2003.68.551 12812343

[tbed13911-bib-0060] Macharia, J., Murithi, R. M., Wainwright, S., Breiman, R. F., Munyua, P., Bloland, P., Njenga, M. K., Githinji, J., Hightower, A., Ithondeka, P. M., Mutonga, D., & Musaa, J. (2010). Rift Valley fever outbreak in livestock in Kenya, 2006–2007. American Journal of Tropical Medicine and Hygiene, 83, 58–64. 10.4269/ajtmh.2010.09-0292 PMC2913503 20682907

[tbed13911-bib-0061] Maggi, R. G., Mascarelli, P. E., Havenga, L. N., Naidoo, V., & Breitschwerdt, E. B. (2013). Co‐infection with *Anaplasma platys*, *Bartonella henselae* and *Candidatus* Mycoplasma haematoparvum in a veterinarian. Parasites & Vectors, 6, 103. 10.1186/1756-3305-6-103 23587235 PMC3637287

[tbed13911-bib-0062] Maina, A. N., Jiang, J., Omulo, S. A., Cutler, S. J., Ade, F., Ogola, E., Feikin, D. R., Kariuki Njenga, M., Cleaveland, S., Mpoke, S., Ng'ang'a, Z., Breiman, R. F., Knobel, D. L. & Richards, A. (2014). High prevalence of *Rickettsia africae* variants in *Amblyomma variegatum* ticks from domestic mammals in rural Western Kenya: Implications for human health. Vector‐Borne and Zoonotic Diseases, 14, 693–702. 10.1089/vbz.2014.1578 25325312 PMC4208559

[tbed13911-bib-0063] Maina, A. N., Klein, T. A., Kim, H.‐C., Chong, S.‐T., Yang, Y. U., Mullins, K., Jiang, J. U., St. John, H., Jarman, R. G., Hang, J., & Richards, A. L. (2017). Molecular characterization of novel mosquito‐borne *Rickettsia* spp. from mosquitoes collected at the Demilitarized Zone of the Republic of Korea. PLoS One, 12(11), e0188327. 10.1371/journal.pone.0188327 29155880 PMC5695765

[tbed13911-bib-0064] Maina, A. N., Luce‐Fedrow, A., Omulo, S., Hang, J., Chan, T.‐C., Ade, F., Jima, D. D., Ogola, E., Ge, H., Breiman, R. F., Njenga, M. K., & Richards, A. L. (2019). Isolation and characterization of a novel *Rickettsia* species (*Rickettsia asembonensis* sp. nov.) obtained from cat fleas (*Ctenocephalides felis*). International Journal of Systematic and Evolutionary Microbiology, 66, 4512–4517. 10.1099/ijsem.0.001382 27506201

[tbed13911-bib-0065] Mans, B. J., Pienaar, R., & Latif, A. A. (2015). A review of *Theileria* diagnostics and epidemiology. International Journal for Parasitology: Parasites and Wildlife, 4(1), 104–118. 10.1016/j.ijppaw.2014.12.006 25830110 PMC4356873

[tbed13911-bib-0066] Mease, L. E., Coldren, R. L., Musila, L. A., Prosser, T., Ogolla, F., Ofula, V. O., Schoepp, R. J., Rossi, C. A., & Adungo, N. (2011). Seroprevalence and distribution of arboviral infections among rural Kenyan adults: A cross‐sectional study. Virology Journal, 8, 371. 10.1186/1743-422X-8-371 21794131 PMC3161961

[tbed13911-bib-0067] Mossel, E. C., Crabtree, M. B., Mutebi, J., Lutwama, J. J., Erin, M., Powers, A. M., & Miller, B. R. (2017). Arboviruses isolated from mosquitoes collected in Uganda, 2008–2012. Journal of Medical Entomology, 54, 1403–1409. 10.1093/jme/tjx120 28874015 PMC5968633

[tbed13911-bib-0068] Mutai, B. K., Wainaina, J. M., Magiri, C. G., Nganga, J. K., Ithondeka, P. M., Njagi, O. N., Jiang, J. U., Richards, A. L., & Waitumbi, J. N. (2013). Zoonotic surveillance for rickettsiae in domestic animals in Kenya. Vector‐Borne and Zoonotic Diseases, 13, 360–366. 10.1089/vbz.2012.0977 23477290

[tbed13911-bib-0069] Mwamuye, M. M., Kariuki, E., Omondi, D., Kabii, J., Odongo, D., Masiga, D., & Villinger, J. (2017). Novel *Rickettsia* and emergent tick‐borne pathogens: A molecular survey of ticks and tick‐borne pathogens in Shimba Hills National Reserve, Kenya. Ticks and Tick‐borne Diseases, 8, 208–218. 10.1016/j.ttbdis.2016.09.002 28011185

[tbed13911-bib-0070] Nabeth, P., Cheikh, D. O., Lo, B., Faye, O., Vall, I. O. M., Niang, M., Wague, B., Diop, D., Diallo, M., Diallo, B., Diop, O. M., & Simon, F. (2004). Crimean‐Congo hemorrhagic fever, Mauritania. Emerging Infectious Diseases, 10, 2143–2149. 10.3201/eid1012.040535 15663851 PMC3323392

[tbed13911-bib-0071] Nakao, R., Qiu, Y., Igarashi, M., Magona, J. W., Zhou, L., Ito, K., & Sugimoto, C. (2013). High prevalence of spotted fever group rickettsiae in *Amblyomma variegatum* from Uganda and their identification using sizes of intergenic spacers. Ticks and Tick‐Borne Diseases, 4(6), 506–512. 10.1016/j.ttbdis.2013.07.001 24331642

[tbed13911-bib-0072] Nakayima, J., Magona, J. W., & Sugimoto, C. (2014). Molecular detection of tick‐borne pathogens in ticks from Uganda. Research, 1, 767. 10.13070/rs.en.1.767

[tbed13911-bib-0073] Ndip, L. M., Bouyer, D. H., Travassos, A. P. A., Rosa, D., Titanji, V. P. K., Tesh, R. B., & Walker, D. H. (2004). Acute spotted fever rickettsiosis among febrile patients, Cameroon. Emerging Infectious Diseases, 10, 3–8. 10.3201/eid1003.020713 PMC3322773 15109409

[tbed13911-bib-0074] Ndip, L. M., Fokam, E. B., Bouyer, D. H., Ndip, R. N., Titanji, V. P. K., Walker, D. H., & Mcbride, J. W. (2004). Detection of *Rickettsia africae* in patients and ticks along the coastal region of Cameroon. American Journal of Tropical Medicine and Hygiene, 71, 363–366. 10.4269/ajtmh.2004.71.363 15381820

[tbed13911-bib-0075] Nijhof, A. M., Bodaan, C., Postigo, M., Nieuwenhuijs, H., Opsteegh, M., Franssen, L., Jebbink, F., & Jongejan, F. (2007). Ticks and associated pathogens collected from domestic animals in the Netherlands. Vector‐Borne and Zoonotic Diseases, 7(4), 585–595. 10.1089/vbz.2007.0130 17979540

[tbed13911-bib-0076] Njiiri, N. E., Bronsvoort, B. M. D. C., Collins, N. E., Steyn, H. C., Troskie, M., Vorster, I., Thumbi, S. M., Sibeko, K. P., Jennings, A., van Wyk, I. C., Mbole‐Kariuki, M., Kiara, H., Poole, E. J., Hanotte, O., Coetzer, K., Oosthuizen, M. C., Woolhouse, M., & Toye, P. (2015). The epidemiology of tick‐borne haemoparasites as determined by the reverse line blot hybridization assay in an intensively studied cohort of calves in western Kenya. Veterinary Parasitology, 210, 69–76. 10.1016/j.vetpar.2015.02.020 25858115 PMC4427107

[tbed13911-bib-0077] Norval, R. A. I., Fivaz, B. H., Lawrence, J. A., & Brown, A. F. (1984). Epidemiology of tick‐borne diseases of cattle in Zimbabwe. Tropical Animal Health and Production, 16, 63–70. 10.1007/bf02239846 6485099

[tbed13911-bib-0078] Nyaruaba, R., Mwaliko, C., Mwau, M., Mousa, S., & Wei, H. (2019). Arboviruses in the East African Community partner states: A review of medically important mosquito‐borne Arboviruses. Pathogens and Global Health, 113(5), 209–228. 10.1080/20477724.2019.1678939 31664886 PMC6882432

[tbed13911-bib-0079] Omondi, D., Masiga, D. K., Fielding, B. C., Kariuki, E., Ajamma, Y. U., Mwamuye, M. M., Ouso, D. O., & Villinger, J. (2017). Molecular detection of tick‐borne pathogen diversities in ticks from livestock and reptiles along the shores and adjacent islands of Lake Victoria and Lake Baringo. Kenya. Frontiers in Veterinary Science, 4, 73. 10.3389/fvets.2017.00073 28620610 PMC5451513

[tbed13911-bib-0080] Oundo, J. W., Villinger, J., Jeneby, M., Ong'amo, G., Otiende, M. Y., Makhulu, E. E., Musa, A. A., Ouso, D. O., & Wambua, L. (2020). Pathogens, endosymbionts, and blood‐meal sources of host‐seeking ticks in the fast‐changing Maasai Mara wildlife ecosystem. PLoS One, 15(8), e0228366. 10.1371/journal.pone.0228366 32866142 PMC7458302

[tbed13911-bib-0081] Papa, A., Tsergouli, K., Tsioka, K., & Mirazimi, A. (2017). Crimean‐Congo hemorrhagic fever: Tick‐host‐virus interactions. Frontiers in Cellular and Infection Microbiology, 7, 213. 10.3389/fcimb.2017.00213 28603698 PMC5445422

[tbed13911-bib-0082] Parola, P., Inokuma, H., Camicas, J., Brouqui, P., & Raoult, D. (2001). Detection and identification of spotted fever group rickettsiae and ehrlichiae in African ticks. Emerging Infectious Diseases, 7(6), 1014–1017. 10.3201/eid0706.010616 11747731 PMC2631901

[tbed13911-bib-0083] Parola, P., Paddock, C. D., Socolovschi, C., Labruna, M. B., Mediannikov, O., Kernif, T., Abdad, M. Y., Stenos, J., Bitam, I., Fournier, P.‐E., & Raoult, D. (2013). Update on tick‐borne rickettsioses around the world: A geographic approach. Clinical Microbiology Reviews, 26, 657–702. 10.1128/CMR.00032-13 24092850 PMC3811236

[tbed13911-bib-0084] Pratt, H. D., & Wiseman, J. S. (1962). Fleas of public health importance and their control: Training guide ‐ insect control series. Public Health Service Publication, 772. https://www.google.com/url?sa=t&rct=j&q=&esrc=s&source=web&cd=&ved=2ahUKEwiKwLudoP_sAhWko3EKHVUBAjQQFjAAegQIAxAC&url=https%3A%2F%2Fstacks.cdc.gov%2Fview%2Fcdc%2F7681%2Fcdc_7681_DS1.pdf&usg=AOvVaw3TkC9ybcxc4FvNUfphRb5I

[tbed13911-bib-0105] Rambaut, A. (2014). FigTree. Version 1.4. 2. University of Edinburgh, Edinburgh, UK. Available at: http://treebioedacuk/software/figtree/ (accessed 27 April 2019).

[tbed13911-bib-0085] Raoult, D., & Roux, V. (1997). Rickettsioses as paradigms of new or emerging infectious diseases. Clinical Microbiology Reviews, 10(4), 694–719. 10.1128/CMR.10.4.694-719.1997 9336669 PMC172941

[tbed13911-bib-0086] Reysenbach, A., Giver, L. J., Wickham, G. S., & Pace, N. R. (1992). Differential amplification of rRNA genes by polymerase chain reaction. Applied and Environmental Microbiology, 58, 3417–3418. 10.1128/AEM.58.10.3417-3418.1992 1280061 PMC183115

[tbed13911-bib-0087] Ringo, A. E., Adjou Moumouni, P. F., Lee, S.‐H., Liu, M., Khamis, Y. H., Gao, Y., Guo, H., Zheng, W., Efstratiou, A., Galon, E. M., Li, J., Tiwananthagorn, S., Inoue, N., Suzuki, H., Thekisoe, O., & Xuan, X. (2018). Molecular detection and characterization of tick‐borne protozoan and rickettsial pathogens isolated from cattle on Pemba Island, Tanzania. Ticks and Tick‐Borne Diseases, 9(6), 1437–1445. 10.1016/j.ttbdis.2018.06.014 30207275

[tbed13911-bib-0088] Roux, V., & Raoult, D. (2000). Phylogenetic analysis of members of the genus *Rickettsia* using the gene encoding the outer membrane protein rOmpB (ompB). International Journal of Systematic and Evolutionary Microbiology, 50, 1449–1455. 10.1099/00207713-50-4-1449 10939649

[tbed13911-bib-0089] Rutherford, J. S., Macaluso, K. R., Smith, N., Zaki, S. R., Paddock, C. D., Davis, J., Peterso, N., Azad, A. F., & Rosenberg, R. (2004). Fatal spotted fever. Emerging Infectious Diseases, 10, 910–913. 10.3201/eid1005.030537 15200829 PMC3323220

[tbed13911-bib-0090] Sang, R., Lutomiah, J., Koka, H., Makio, A., Chepkorir, E., Ochieng, C., Yalwala, S., Mutisya, J., Musila, L., Richardson, J. H., Miller, B. R. & Schnabel, D. (2011). Crimean‐congo hemorrhagic fever virus in hyalommid ticks, northeastern Kenya. Emerging Infectious Diseases, 17, 1502–1505. 10.3201/eid1708.102064 21801635 PMC3381575

[tbed13911-bib-0091] Sang, R., Onyango, C., Gachoya, J., Mabinda, E., Konongoi, S., Ofula, V., Dunster, L., Okoth, F., Coldren, R., Tesh, R., Travassos da Rosa, A., Finkbeiner, S., Wang, D., Crabtree, M., & Miller, B. (2006). Tickborne arbovirus surveillance in market livestock, Nairobi. Kenya. Emerging Infectious Diseases, 12, 1074–1080. 10.3201/eid1207.060253 16836823 PMC3291068

[tbed13911-bib-0092] Sergeant, E. S. G. (2018). Epitools epidemiological calculators. Ausvet. Retrieved from http://epitools.ausvet.com.au

[tbed13911-bib-0093] Simpson, D. I. H., Knight, E. M., Courtois, G., Williams, M. C., Weinbren, M. P., & Kibukamusoke, J. W. (1967). Congo virus: A hitherto undescribed virus occurring in Africa. Part I. Human isolations‐clinical notes. East African Medical Journal, 44, 87–92. 6040759

[tbed13911-bib-0094] Singh, N. K., Haque, M., Jyoti, Rath, S. S., & Ghosh, S. (2011). First report of *Ctenocephalides felis* felis infestation of buffalo calves in Punjab, India. Journal of Parasitic Diseases, 35(2), 235–236. 10.1007/s12639-011-0038-3. 23024513 PMC3235394

[tbed13911-bib-0095] Sparagano, O. A., Allsopp, M. T., Mank, R. A., Rijpkema, S. G., Figueroa, J. V., & Jongejan, F. (1999). Molecular detection of pathogen DNA in ticks (Acari: Ixodidae): A review. Experimental & Applied Acarology, 23(12), 929–960. 10.1023/a:1006313803979 10737729

[tbed13911-bib-0096] Spengler, J. R., Bergeron, É., & Rollin, P. E. (2016). Seroepidemiological studies of Crimean‐Congo hemorrhagic fever virus in domestic and wild animals. PLoS Neglected Tropical Diseases, 10(1), e0004210. 10.1371/journal.pntd.0004210 26741652 PMC4704823

[tbed13911-bib-0097] Teshale, S., Geysen, D., Ameni, G., Asfaw, Y., & Berkvens, D. (2015). Improved molecular detection of *Ehrlichia* and *Anaplasma* species applied to *Amblyomma* ticks collected from cattle and sheep in Ethiopia. Ticks and Tick‐Borne Diseases, 6(1), 1–7. 10.1016/j.ttbdis.2014.04.023 25438799

[tbed13911-bib-0098] Villinger, J., Mbaya, M. K., Ouso, D., Kipanga, P. N., Lutomiah, J., & Masiga, D. K. (2017). Arbovirus and insect‐specific virus discovery in Kenya by novel six genera multiplex high‐resolution melting analysis. Molecular Ecology Resources, 17(3), 466–480. 10.1111/1755-0998.12584 27482633

[tbed13911-bib-0100] Wesonga, F. D., Kitala, P. M., Gathuma, J. M., Njenga, M. J., & Ngumi, P. N. (2010). An assessment of tick‐borne diseases constraints to livestock production in a smallholder livestock production system in Machakos District, Kenya. Livestock Research for Rural Development, 22(6), 111.

[tbed13911-bib-0101] WHO (2018). Weekly bulletin on outbreaks and other emergencies. Week 26: June 2018. World Health Orgaisation. Retrieved from https://apps.who.int/iris/bitstream/handle/10665/272981/OEW26‐2329062018.pdf.

[tbed13911-bib-0102] Williams, C. J., & Moffitt, C. M. (2001). A critique of methods of sampling and reporting pathogens in populations of fish. Journal of Aquatic Animal Health, 13(4), 300–309. 10.1577/1548-8667(2001)013<0300:ACOMOS>2.0.CO;2

[tbed13911-bib-0103] Woolhouse, M. E. J., Thumbi, S. M., Jennings, A., Chase‐Topping, M., Callaby, R., Kiara, H., Oosthuizen, M. C., Mbole‐Kariuki, M. N., Conradie, I., Handel, I. G., Poole, E. J., Njiiri, E., Collins, N. E., Murray, G., Tapio, M., Auguet, O. T., Weir, W., Morrison, W. I., Kruuk, L. E. B., … Toye, P. G. (2015). Co‐infections determine patterns of mortality in a population exposed to parasite infection. Science Advances, 1(2), e1400026. 10.1126/sciadv.1400026 26601143 PMC4643819

[tbed13911-bib-0104] Zhong, J., Jasinskas, A., & Barbour, A. G. (2007). Antibiotic treatment of the tick vector *Amblyomma americanum* reduced reproductive fitness. PLoS One, 2(5), e405. 10.1371/journal.pone.0000405 17476327 PMC1852332

