# Supplementary material for: Tick‐borne pathogens, including Crimean‐Congo haemorrhagic fever virus, at livestock markets and slaughterhouses in western Kenya
Source: Transbound Emerg Dis. 2020 Dec 5;68(4):2429–45. doi: 10.1111/tbed.13911 (PMC8359211; doi:10.1111/tbed.13911)
Supplement: Supplementary file 1 — Fig S1 [file TBED-68-2429-s003.docx]

**
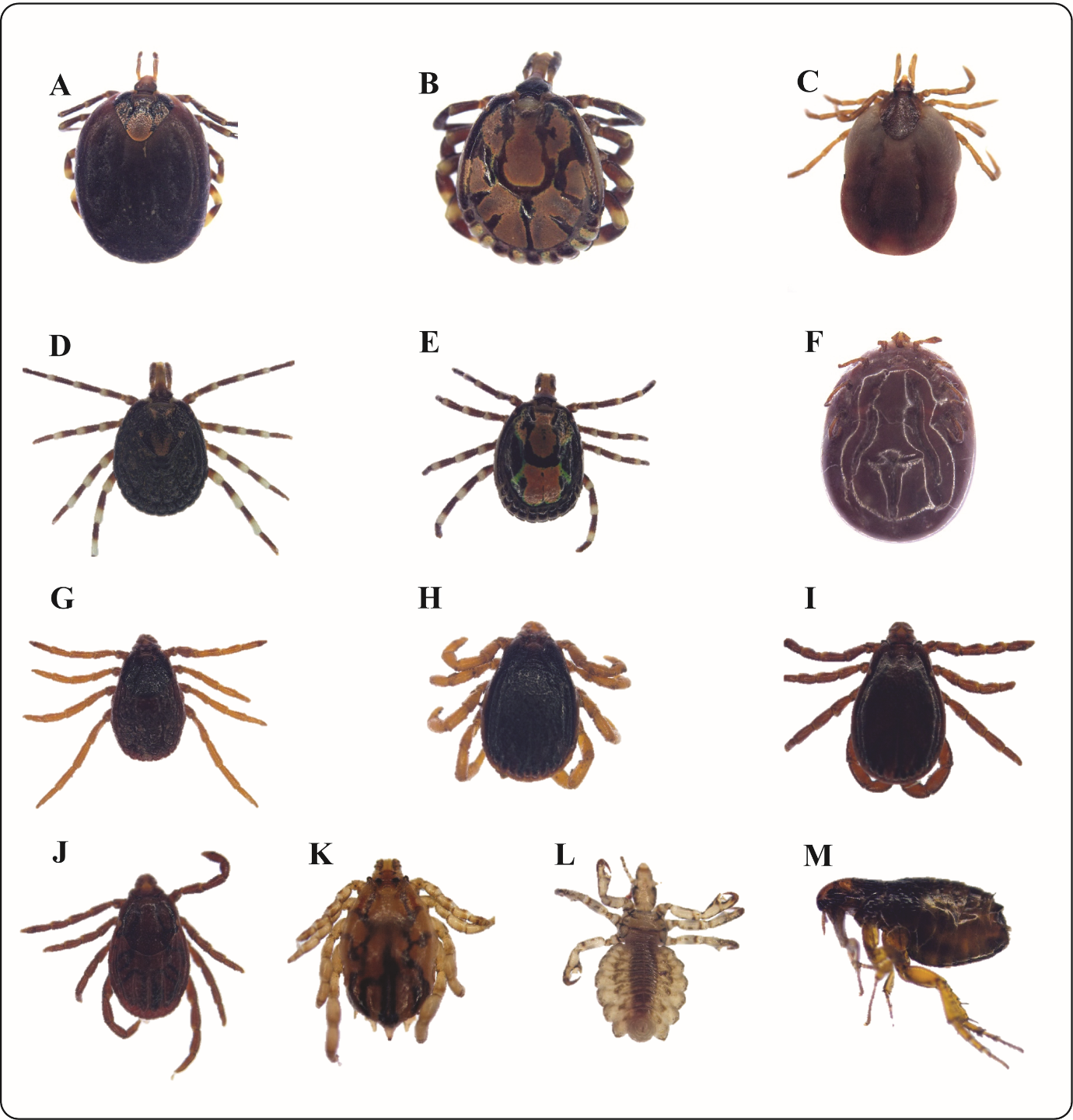
**

**Supplementary Figure 1. Photographs of representative specimens of vectors collected from livestock at livestock markets and slaughterhouses:** A. *Am. gemma* female; B. *Am. gemma* male; C. *Amblyomma* sp. nymph; D. *Am variegatum* female; E. *Am variegatum* male; F. *Haemaphysalis* sp.; G. *Rhipicephalus evertsi* female and male; H. *Rh. evertsi* male; I. *Rh. appendiculatus* male; J. *Rh. appendiculatus* female; K. *Rh. decoloratus*; L. *Haematopinus suis*; M. *Ctenocephalides felis***.**
