# Supplementary material for: Tick‐borne pathogens, including Crimean‐Congo haemorrhagic fever virus, at livestock markets and slaughterhouses in western Kenya
Source: Transbound Emerg Dis. 2020 Dec 5;68(4):2429–45. doi: 10.1111/tbed.13911 (PMC8359211; doi:10.1111/tbed.13911)
Supplement: Supplementary file 2 — Fig S2 [file TBED-68-2429-s005.docx]

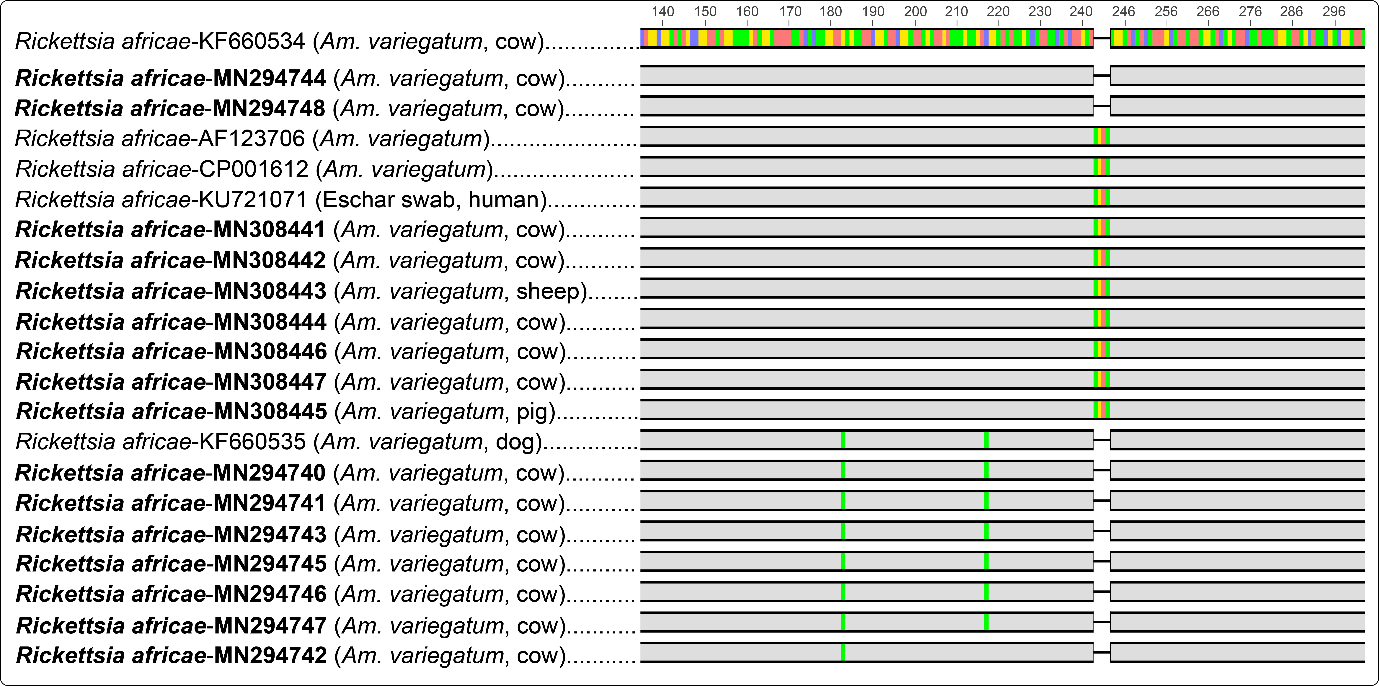


**Supplementary Figure 2.** **Partial *omp*B gene sequences of *Rickettsia africae* obtained from this study aligned with GenBank reference sequences.** Accession numbers of sequences from this study are in bold. Note the deletion mutation of a 4-base pair motif and several base substitutions in the sequences. Red = Adenine; Blue = Cytosine; Green = Thymine; Yellow = Guanine; Grey = consensus with *R. africae* reference
