# Supplementary material for: Tick‐borne pathogens, including Crimean‐Congo haemorrhagic fever virus, at livestock markets and slaughterhouses in western Kenya
Source: Transbound Emerg Dis. 2020 Dec 5;68(4):2429–45. doi: 10.1111/tbed.13911 (PMC8359211; doi:10.1111/tbed.13911)
Supplement: Supplementary file 3 — Table S1 [file TBED-68-2429-s002.docx]

**Supplementary Table 1**: Details of primers used in the molecular identification of ticks and screening for vector-borne pathogens

| **Primer name** | **Target gene** | **Primer sequence (5’ – 3’)** | **Product size (bp)** | **Citation** |
| --- | --- | --- | --- | --- |
| LepF | Tick CO1 | ATTCAACCAATCATAAAGATATTGG | 658 | Hebert et al., 2004 |
| Lep R |  | TAAACTTCTGGATGTCCAAAAAATCA |  |  |
| ITS2 F | Tick ITS2 | CGAGACTTGGTGTGAATTGCA | 920-1850 | Chitimia et al., 2009 |
| ITS2 R |  | TCCCATACACCACATTTCCCG |  |  |
| F-RMI16S | Tick 16S rRNA | AATTGCTGTAGTATTTTGAC | 450 | Brahma et al., 2014 |
| R-RMI16S |  | TCTGAACTCAGATCAAGTAG |  |  |
| Phlebo JV3a F | *Phlebovirus NP(S-segment* | AGTTTGCTTATCAAGGGTTTGATGC |  | Villinger et al., 2017 |
| Phlebo JV3b F |  | GAGTTTGCTTATCAAGGGTTTGACC |  |  |
| Phlebo JV3 R |  | CCGGCAAAGCTGGGGTGCAT |  |  |
| Nairo L 1a F | *Nairovirus RdRp* | TCTCAAAGATATCAATCCCCCCITTACCC |  | Villinger et al., 2017 |
| Nairo L 1b F |  | TCTCAAAGACATCAATCCCCCTTWTCCC |  |  |
| Nairo L 1a R |  | CTATRCTGTGRTAGAAGCAGTTCCCATC |  |  |
| Nairo L 1b R |  | GCAATACTATGATAAAAACAATTMCCATCAC |  |  |
| Nairo L 1c R |  | CAATGCTGTGRTARAARCAGTTGCCATC |  |  |
| Nairo L 1d R |  | GCAATGCTATGGTAGAAACAGTTTCCATC |  |  |
| Nairo L 1e R |  | CRAKGCTGTGGTAAAAGCAGTTRCCATC |  |  |
| Bunyagroup F | *Orthobunyavirus NP (S-segment)* | CTGCTAACACCAGCAGTACTTTTGAC |  | Lambert and Lanciotti, 2009 |
| Bunyagroup R |  | TGGAGGGTAAGACCATCGTCAGGAACTG |  |  |
| Vir 2052 F | *Alphavirus NSP4* | TGGCGCTATGATGAAATCTGGAATGTT |  | Eshoo et al., 2007 |
| Vir 2052 R |  | TACGATGTTGTCGTCGCCGATGAA |  |  |
| Flavi JV2a F | *Flavivirus NSP5* | AGYMGHGCCATHTGGTWCATGTGG |  | Villinger et al., 2017 |
| Flavi JV2b F |  | AGCCGYGCCATHTGGTATATGTGG |  |  |
| Flavi JV2c F |  | AGYCGMGCAATHTGGTACATGTGG |  |  |
| Flavi JV2d F |  | AGTAGAGCTATATGGTACATGTGG |  |  |
| Flavi JV2a R |  | GTRTCCCADCCDGCDGTRTCATC |  |  |
| Flavi JV2b R |  | GTRTCCCAKCCWGCTGTGTCGTC |  |  |
| Thogoto S6 F | *Thogoto* virus *M-segment* | GATGACAGYCCTTCTGCAGTGGTGT |  | Villinger et al., 2017 |
| Thogoto S6 R |  | RACTTTRTTGCTGACGTTCTTGAGGAC |  |  |
| Dhori F | *Dhori* virus *NP* | CGAGGAAGAGCAAAGGAAAG |  | Villinger et al., 2017 |
| Dhori R |  | GTGCGCCCCTCTGGGGTTT |  |  |
| Nairo 6942 | *Nairovirus RdRp* | ATGATTGCIAAYAGIAAYTTYAA | 434 | Honig et al., 2004 |
| Nairo 7385 |  | ACAGCARTGIATIGGICCCCAYTT |  |  |
| AnaplasmaJV F | *Anaplasma* 16R rRNA | CGGTGGAGCATGTGGTTTAATTC | 300 | Mwamuye et al., 2017 |
| AnaplasmaJV R |  | CGRCGTTGCAACCTATTGTAGTC |  |  |
| EhrlichiaJV F | *Ehrlichia* 16S rRNA | GCAACCCTCATCCTTAGTTACCA | 300 | Mwamuye et al., 2017 |
| EhrlichiaJV R |  | TGTTACGACTTCACCCTAGTCAC |  |  |
| 16SD | *Anaplasma*16S rRNA | GGTACCYACAGAAGAAGTCC | 1090 | Parola et al., 2000 Edwards et al., 1989 |
| pH1522 |  | AAGGAGGTGATCCAGCCGCA |  | Reysenbach et al., 1992 |
| pH1492 |  | GGCTACCTTGTTACGACTT | 1030 |  |
| Rick-F | *Rickettsia* 16S rRNA | GAACGCTATCGGTATGCTTAACACA | 364 | Nijhof et al., 2007 |
| Rick-R |  | CATCACTCACTCGGTATTGCTGGA |  |  |
| ompB 120–2788 | *Rickettsia* ompB | AAACAATAATCAAGGTACTGT | 856 | Roux and Raoult, 2000 |
| ompB 120–3599 |  | TACTTCCGGTTACAGCAAAGT |  |  |
| RLB-F | *Theileria/Babesia* 18S rRNA | GAGGTAGTGACAAGAAATAACAATA | 450 | Georges et al., 2001 |
| RLB-R |  | TCTTCGATCCCCTAACTTTC |  |  |
|  |  |  |  |  |
