# Supplementary material for: Tick‐borne pathogens, including Crimean‐Congo haemorrhagic fever virus, at livestock markets and slaughterhouses in western Kenya
Source: Transbound Emerg Dis. 2020 Dec 5;68(4):2429–45. doi: 10.1111/tbed.13911 (PMC8359211; doi:10.1111/tbed.13911)
Supplement: Supplementary file 4 — Table S2 [file TBED-68-2429-s004.docx]

**Supplementary table 2**: Numbers of vertebrate species from which vectors were collected in the different livestock markets and slaughterhouses in Western Kenya between 6/04/2017 and 7/06/2018

|  |  |  |  | **No. of hosts sampled from** | | | |
| --- | --- | --- | --- | --- | --- | --- | --- |
| **Study area** | **Vector species** | **Number (N)** | **No. Pools** | **Cattle** | **Goat** | **Sheep** | **Pig** |
| Amerikwa | *Rhipicephalus* sp*.* | 4 | 2 | 1 |  |  |  |
| Amukura | *Am. variegatum* | 7 | 6 | 6 |  |  |  |
|  | *Rhipicephalus* sp*.* | 1 | 1 | 1 |  |  |  |
|  | *Rh. decoloratus* | 1 | 1 | 1 |  |  |  |
|  | *Rh. evertsi* | 3 | 1 | 1 |  |  |  |
|  | *Rh. appendiculatus* | 6 | 3 | 2 |  |  |  |
| Angurai | *Rh. decoloratus* | 11 | 5 | 2 |  |  |  |
|  | *Rh. appendiculatus* | 9 | 4 | 2 |  |  |  |
| Bumala | *Haemaphysalis* sp*.* | 1 | 1 | 1 |  |  |  |
| Butula | *Am. variegatum* | 27 | 16 | 11 |  |  |  |
|  | *Rh. decoloratus* | 8 | 5 | 3 |  |  |  |
|  | *Rhipicephalus* sp. | 2 | 2 | 2 |  |  |  |
|  | *Amblyomma* sp. | 1 | 1 |  | 1 |  |  |
|  | *H. suis* | 5 | 2 |  |  |  | 2 |
| Chwele | *Am. variegatum* | 5 | 3 | 3 |  |  |  |
|  | *A. gemma* | 3 | 3 | 1 |  |  |  |
|  | *Rh. decoloratus* | 2 | 1 | 1 |  |  |  |
|  | *Rh. evertsi* | 1 | 1 | 1 |  |  |  |
| Funyula | *Am. variegatum* | 11 | 7 | 3 |  |  | 3 |
|  | *Rh. appendiculatus* | 6 | 4 | 2 |  |  | 1 |
|  | *Rh. appendiculatus nymph* | 1 | 1 | 1 |  |  |  |
|  | *Rhipicephalus* sp*.* | 2 | 2 | 2 |  |  |  |
|  | *Rh. evertsi* | 3 | 1 |  | 1 |  |  |
|  | *H. suis* | 6 | 4 |  |  |  | 4 |
| Harambe | *Am. variegatum* | 2 | 2 | 2 |  |  |  |
|  | *Rh. decoloratus* | 2 | 1 | 1 |  |  |  |
|  | *Rh. microplus* | 2 | 1 | 1 |  |  |  |
|  | *Rhipicephalus* sp*.* | 1 | 1 | 1 |  |  |  |
| Ikolomani | *Am. variegatum* | 7 | 5 | 3 |  | 1 |  |
|  | *Rh. decoloratus* | 8 | 6 | 5 |  | 1 |  |
|  | *Rh. microplus* | 4 | 1 | 1 |  |  |  |
|  | *Rh. evertsi* | 4 | 2 |  | 1 |  |  |
|  | *Rh. appendiculatus* | 4 | 2 | 2 |  |  |  |
|  | *Rhipicephalus* sp*.* | 7 | 4 | 4 |  |  |  |
|  | *H. suis* | 6 | 4 |  |  |  | 4 |
| Kimilili | *Am. variegatum* | 6 | 4 | 3 |  |  |  |
|  | *Rh. decoloratus* | 41 | 24 | 16 |  |  |  |
|  | *Rh. appendiculatus* | 1 | 1 | 1 |  |  |  |
|  | *Rh. evertsi* | 3 | 3 | 2 | 1 |  |  |
|  | *Rhipicephalus* sp*.* | 23 | 18 | 12 | 1 | 1 |  |
| Koyonzo | *Am. variegatum* | 9 | 7 | 4 |  |  |  |
|  | *Rh. decoloratus* | 3 | 3 | 3 |  |  |  |
|  | *Rh. evertsi* | 1 | 1 | 1 |  |  |  |
| Lubao | *Am. variegatum* | 3 | 3 | 1 |  |  | 1 |
|  | *Amblyomma* sp. | 1 | 1 | 1 |  |  |  |
|  | *Rh. decoloratus* | 29 | 20 | 13 |  |  |  |
|  | *Rh. evertsi* | 3 | 2 | 2 |  |  |  |
|  | *Rhipicephalus* sp*.* | 8 | 8 | 5 |  |  |  |
|  | *H. suis* | 5 | 4 |  |  |  | 4 |
|  | *Rh. appendiculatus* | 1 | 1 | 1 |  |  |  |
| Malaba | *Am. variegatum* | 13 | 10 | 6 |  |  |  |
|  | *Rh. decoloratus* | 3 | 2 | 1 | 1 |  |  |
|  | *Rh. evertsi* | 1 | 1 | 1 |  |  |  |
|  | *Rh. appendiculatus* | 2 | 2 | 2 |  |  |  |
| Myanga | *Am. variegatum* | 22 | 15 | 11 |  |  |  |
|  | *Amblyomma* sp. | 1 | 1 | 1 |  |  |  |
|  | *Rh. decoloratus* | 9 | 6 | 5 |  |  |  |
|  | *Rh. appendiculatus* | 18 | 11 | 8 |  |  |  |
|  | *Rh. evertsi* | 5 | 5 | 3 |  |  |  |
| Shinyalu | *Am. variegatum* | 8 | 8 | 7 |  |  |  |
|  | *Rh. decoloratus* | 31 | 22 | 15 |  |  |  |
|  | *Rh. appendiculatus* | 4 | 2 | 2 |  |  |  |
|  | *Amblyomma* sp. | 2 | 2 | 2 |  |  |  |
|  | *H. suis* | 6 | 3 |  |  |  | 3 |
|  | *Rhipicephalus* sp*.* | 10 | 9 | 5 | 1 |  |  |
| Webuye | *Am. variegatum* | 1 | 1 | 1 |  |  |  |
|  | *Rh. decoloratus* | 14 | 10 | 7 |  |  |  |
|  | *Rhipicephalus* sp. | 10 | 6 | 4 |  | 1 |  |
