# Supplementary material for: Tick‐borne pathogens, including Crimean‐Congo haemorrhagic fever virus, at livestock markets and slaughterhouses in western Kenya
Source: Transbound Emerg Dis. 2020 Dec 5;68(4):2429–45. doi: 10.1111/tbed.13911 (PMC8359211; doi:10.1111/tbed.13911)
Supplement: Supplementary file 5 — Table S3 [file TBED-68-2429-s001.docx]

**Supplementary Table 3:** Occurrence of vector-borne pathogens isolated from ticks and lice from livestock markets and slaughterhouses

| Pathogen detected | Vector species | Livestock market/slaughterhouse | Vertebrate species | GenBank accessions |
| --- | --- | --- | --- | --- |
| *Anaplasma platys* | *Rhipicephalus decoloratus, Rhipicephalus sp., Rh. appendiculatus* | Lubao, Kimilili, Funyula, Shinyalu, Butula, Angurai, Myanga, Webuye | Cattle | MN266939-MN266941 |
| *A. ovis* | *Rhipicephalus sp., Rh. evertsi, Rh. decoloratus* | Shinyalu, Ikolomani, Kimilili, Webuye, Amukura | Goat, cattle | MN266936-MN266938 |
| *A. marginale* | *Rh. decoloratus, Rhipicephalus sp*. | Kimilili, Shinyalu, Lubao | Cattle | MN266931-MN266935 |
| *Rickettsia africae* | *Amblyomma variegatum, Am. gemma, Rh. appendiculatus, Haematopinus suis, Rh. decoloratus, Rhipicephalus* sp. | Myanga, Ikolomani, Amukura, Malaba, Funyula, Lubao, Shinyalu, Butula, Chwele, Kimilili, Koyonzo, Angurai, Amerikwa, Harambe | Cattle, pig, goat, sheep | MN294740-MN294749 |
| *Babesia caballi* | *Am. variegatum* | Myanga, Malaba, Ikolomani, Koyonzo, Funyula, Butula | Cattle, sheep, pig | MN294721-MN294723 |
| *B. bigemina* | *Rh. decoloratus, Am. variegatum, Rh. appendiculatus* | Angurai, Amukura | Cattle | MN294720 |
| *Hepatozoon canis* | *Rh. decoloratus* | Kimilili | Cattle | MN294724 |
| *Theileria mutans* | *Rh. decoloratus, Rhipicephalus sp. Am. variegatum, Rh. appendiculatus* | Shinyalu, Kimilili, Lubao, Butula, Koyonzo, Myanga, Webuye, Ikolomani | Cattle | MN294725-MN294729 |
| *T. parva* | *Rhipicephalus* sp. | Funyula | Cattle | MN294730 |
| *T. taurotragi* | *Rh. decoloratus, Rhipicephalus sp., Rh. appendiculatus* | Kimilili, Lubao, Angurai | Cattle | MN294731-MN294732 |
| *T. velifera* | *Am. variegatum, Rhipicephalus* sp. | Kimilili, Shinyalu, Lubao | Cattle | MN294733-MN294734 |
| CCHF virus | *Rh. decoloratus, Rhipicephalus* sp. | Shinyalu, Lubao | Cattle | MN267048-MN267049 |
| *Coxiella* endosymbionts | All tick species except *Haemaphysalis* sp. | All livestock markets and slaughterhouses | All livestock species | MN262071-MN262076; MN266946-MN266948; MN266922-MN266928 |
